# Single-zinc vacancy unlocks high-rate H_2_O_2_ electrosynthesis from mixed dioxygen beyond Le Chatelier principle

**DOI:** 10.1038/s41467-024-48256-7

**Published:** 2024-05-16

**Authors:** Qi Huang, Baokai Xia, Ming Li, Hongxin Guan, Markus Antonietti, Sheng Chen

**Affiliations:** 1 Key Laboratory for Soft Chemistry and Functional Materials, School of Chemistry and Chemical Engineering, Nanjing University of Science and Technology, Ministry of Education, Nanjing, 210094 China; 2 Max Planck Institute of Colloids and Interfaces, Potsdam, 214476 Germany

**Keywords:** Electrocatalysis, Electrocatalysis, Electrocatalysis

## Abstract

Le Chatelier’s principle is a basic rule in textbook defining the correlations of reaction activities and specific system parameters (like concentrations), serving as the guideline for regulating chemical/catalytic systems. Here we report a model system breaking this constraint in O_2_ electroreduction in mixed dioxygen. We unravel the central role of creating single-zinc vacancies in a crystal structure that leads to enzyme-like binding of the catalyst with enhanced selectivity to O_2_, shifting the reaction pathway from Langmuir-Hinshelwood to an upgraded triple-phase Eley-Rideal mechanism. The model system shows minute activity alteration of H_2_O_2_ yields (25.89~24.99 mol g_cat_^−1^ h^−1^) and Faradaic efficiencies (92.5%~89.3%) in the O_2_ levels of 100%~21% at the current density of 50~300 mA cm^−2^, which apparently violate macroscopic Le Chatelier’s reaction kinetics. A standalone prototype device is built for high-rate H_2_O_2_ production from atmospheric air, achieving the highest Faradaic efficiencies of 87.8% at 320 mA cm^−2^, overtaking the state-of-the-art catalysts and approaching the theoretical limit for direct air electrolysis (~345.8 mA cm^−2^). Further techno-economics analyses display the use of atmospheric air feedstock affording 21.7% better economics as comparison to high-purity O_2_, achieving the lowest H_2_O_2_ capital cost of 0.3 $ Kg^−1^. Given the recent surge of demonstrations on tailoring chemical/catalytic systems based on the Le Chatelier’s principle, the present finding would have general implications, allowing for leveraging systems “beyond” this classical rule.

## Abstract (web summary)

The principle of Le Chatelier is a fundamental concept in textbooks, serving as a guiding principle for controlling chemical and catalytic systems. In this study, the authors present an oxygen electroreduction system based on a single zinc vacancy catalyst, which operates in a manner that extends “beyond” Le Chatelier’s principle.

## Introduction

Two-electron oxygen reduction reaction (ORR) offers an alternative/supplementary route for traditional anthraquinone process, enabling the distributed on-demand production of H_2_O_2_ under ambient condition^1–3^:

1 $${{{{{\rm{O}}}}}}_{2}+{{{{{\rm{H}}}}}}_{2}{{{{\rm{O}}}}}+2{{{{{\rm{e}}}}}}^{-}\to {{{{{\rm{HO}}}}}}_{2}^{-}+{{{{{\rm{OH}}}}}}^{-} \quad\quad({E}^{{{{{\rm{o}}}}}}=0.06\,{{{{\rm{V}}}}}\,{{{{\rm{vs}}}}}.\,{{{{\rm{SHE}}}}},\,{{{{\rm{in}}}}}\,{{{{\rm{alkaline}}}}}\,{{{{\rm{media}}}}})$$

2 $${{{{{\rm{O}}}}}}_{2}+2{{{{{\rm{H}}}}}}^{+}+2{{{{{\rm{e}}}}}}^{-}\to {{{{{\rm{H}}}}}}_{2}{{{{{\rm{O}}}}}}_{2} \quad\quad\quad ({E}^{{{{{\rm{o}}}}}}=0.68\,{{{{\rm{V}}}}}\,{{{{\rm{vs}}}}}.\,{{{{\rm{SHE}}}}},\,{{{{\rm{in}}}}}\,{{{{\rm{neutral}}}}}/{{{{\rm{acidic}}}}}\,{{{{\rm{media}}}}})$$

Research into viable ORR catalytic systems has received growing interest^4–9^, but there are several long-standing challenges that prevent ORR from implemented on commercial scale, including but not limited to the competition of four-electron pathway^6^, the low solubility of O_2_ in electrolytes^5^, the decomposition of H_2_O_2_ product^5^ and long-term system durability^7^.

Another critical issue relatively unexplored is the impact of O_2_ levels on the synthetic system. Since the first report of ORR a century ago^10^, it seemed common conception that the alteration of O_2_ concentrations by diluents (like N_2_) would disturb the equilibria of the reaction, and according to the Le Chatelier principle^11^, resulting in performance losses.

Nevertheless, the exposure of a catalytic system to common diluents or impurities, especially under practically applied conditions, is almost unavoidable. An ORR electrochemical cell is composed of cathodic/anodic reactions separated by membranes and connected by electrolytes^5^. The crossover of gases/products derived from ORR or its coupled processes (like N_2_/CO_2_ from Fenton process^12,13^, C_2_H_4_ from cascade synthesis^14^ and Cl_2_ from anodic seawater oxidation^15^) results in the high dilution of O_2_. Ambient external gases may also directly transfer into the cathodic chamber to contact with ORR catalysts upon the membrane deactivation/device aging after long-term operation^3^. A promising strategy for H_2_O_2_ synthesis is to mimic natural enzyme superoxide dismutase, which generates H_2_O_2_ in full air atmosphere. The air composed of O_2_ and N_2_ is flushed into the electrochemical cells and dissolves near the reaction centers in minute amounts in the aqueous electrolyte. Under all the above scenario, the O_2_ in mixed gas media quickly reacts away, and the decrease of O_2_ levels usually reduces the initial reaction rates/equilibrium following the Le Chatelier principle^11^, resulting in activity decay of H_2_O_2_ yields and Faradic efficiencies. The reaction is dependent on electrode substrate concentration and its diffusive replacement rate.

To the best of our knowledge, the majority of contributions still focus on the conversion of high-purity O_2_ (>99.99%) prepared from air via the complex steps of separation, purification, compression and transportation, which would increase the production cost of H_2_O_2_. Developing a system that could achieve excellent ORR activities in a wide range of O_2_ concentrations represent a significant progress toward practical applications. Importantly, this study also covers a typical problem of electrochemistry, i.e., it is difficult to exclude the diluents or impurities such as the crossover of gases/products in electrochemical systems. How is the partly complex dependence of the reaction rates on the gas partial pressure to be modeled and harnessed, so the system achieves improved activities?

The Eley-Rideal^16,17^ and Langmuir-Hinshelwood mechanisms^18^ are well-documented reaction models describing the adsorption and conversion at chemical/catalytic interfaces. The Eley-Rideal mechanism shows promise for ORR at high O_2_ dilution because of only requires one of the reactant molecules to bind with the active site of catalysts followed by reacting directly with the other molecules from the volume phase. This is different from the Langmuir-Hinshelwood mechanism requiring both reactant molecules adsorbed onto the active sites of catalysts. The conventional Eley-Rideal mechanism only models the reactions at binary gas-solid interfaces. For gas-involving electrochemistry in liquids, like ORR, this model needs to be upgraded to include triple-phase interfaces, as high-rate ORR occurs at the ternary boundaries of O_2_ gas/aqueous electrolytes/catalysts. Very recently, single-atom catalysts have been extensively reported in chemical/catalytic systems because of their excellent properties such as high metal utilizations and strong metal-support interactions^6,12^. While single-atom vacancy catalysts, the counterpart of single-atom catalysts except of using vacancies, have been still underexplored.

In this work, we find the reference zinc oxide (ZnO) crystal promoting ORR through Langmuir-Hinshelwood mechanism, which shifts to the Eley-Rideal mechanism after introducing single-zinc vacancies (denoted as Eley-Rideal-mechanism-ZnO or ER-ZnO). These vacancies allow for an enzyme-like binding of the catalyst with high selectivity to O_2_ in mixed dioxygen media. The catalyst model shows practical no dependence of reaction rates on the O_2_ partial pressure, and O_2_ adsorption and availability are not the rate-determining step, even at low O_2_ levels, i.e., the Le Chatelier’s principle is out of importance in the system.

## Results

### Limitation of ZnO catalyst in mixed dioxygen electroreduction

**Fig. 1:**
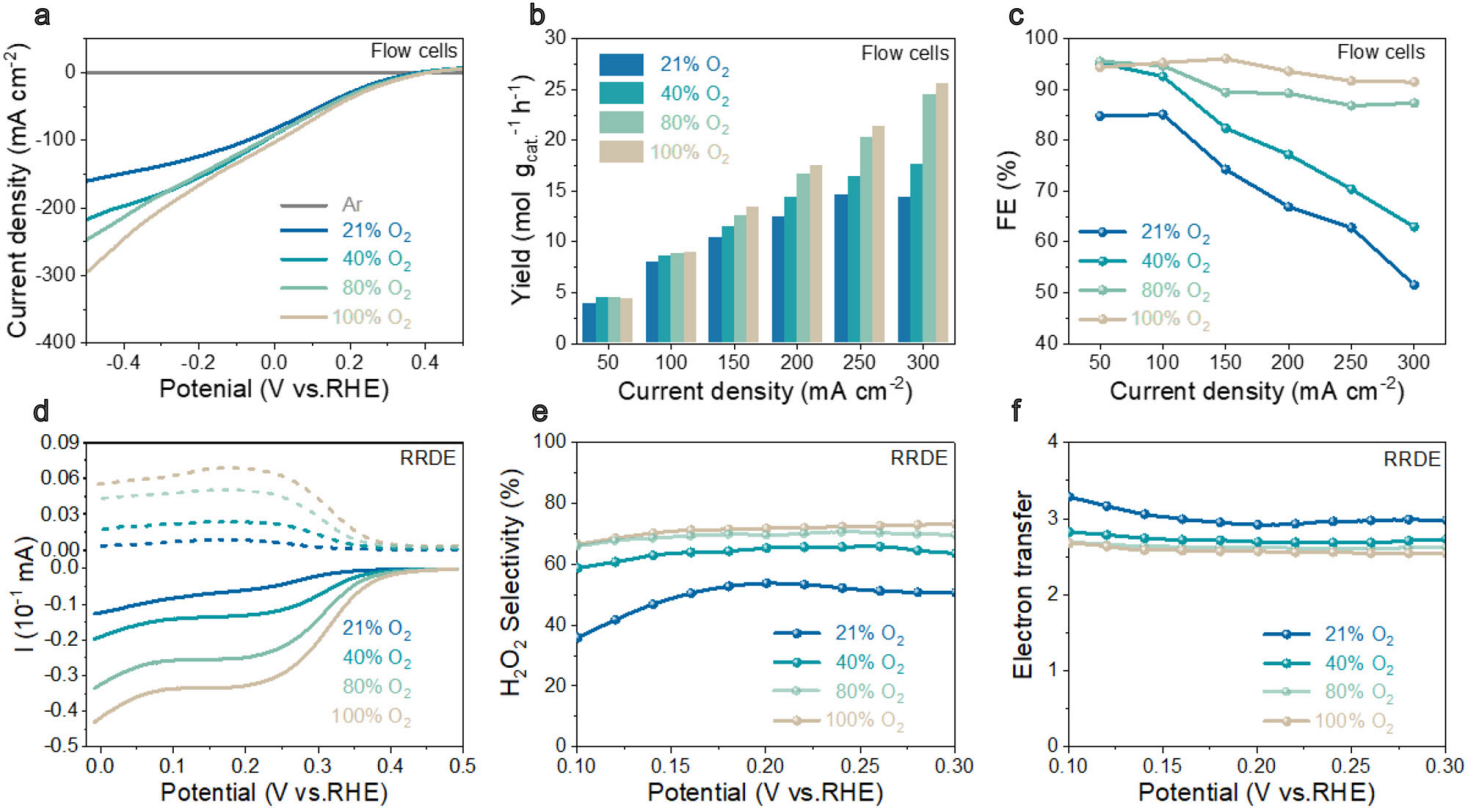
The limitation of reference ZnO catalyst for oxygen electroreduction to H_2_O_2_ in mixed O_2_ media (21%, 40%, 80%, and 100% O_2_ in O_2_/N_2_ mixtures). **a** LSV curves tested in flow cells. **b** H_2_O_2_ yield s tested in flow cells. **c** H_2_O_2_ Faradaic efficiencies tested in flow cells. **d** LSV polarization curves on RRDE at a rotation speed of 1600 rpm and scan rate of 5 mV s^−1^, where the H_2_O_2_ currents were recorded on the ring electrode at a constant potential of 1.2 V (vs. RHE). **e** H_2_O_2_ selectivity calculated on the basis of RRDE. **f** Electron transfer number (*n*) calculated on the basis of RRDE.

The reference ZnO catalyst was synthesized by the controlled hydrolysis of zinc acetate (Supplementary Fig. 1), which was loaded onto gas-diffusion electrodes assembled in standard flow-type electrolytic cells for evaluating the ORR activities in mixed O_2_ media (100%, 80%, 40%, and 21% O_2_ in O_2_/N_2_ mixtures; Fig. 1a). The ORR activities were quantified by chronoamperometric tests at different current densities (Figs. 1b, c and Supplementary Fig. 2). Lowering O_2_ concentrations from 100% to 21% with this reference ZnO catalyst, the H_2_O_2_ yields and Faradaic efficiencies show a significant decay at current densities between 50 and 300 mA cm^−2^. More specifically at 300 mA cm^−2^, the H_2_O_2_ yields are 25.6, 24.4, 17.6, and 14.4 mol g_cat_^−1^ h^−1^ in 100%, 80%, 40%, and 21% O_2_, respectively. This reflects the above-discussed oxygen depletion at the active centers, that is, the ORR turns transport limited at the triple-phase interfaces. Analogously, the H_2_O_2_ Faradaic efficiencies decrease from 91.5%, 87.3%, 63.0% to 51.5% for 100%, 80%, 40%, and 21% O_2_, respectively (Fig. 1c). This result is further consistent to rotation ring disk electrode (RRDE) measurements in generator-collector mode (Fig. 1d–f), showing a declined H_2_O_2_ selectivity (from 70% to 49%) and elevated electron transfer numbers (from 2.6 to 3.2) with lowering O_2_ concentrations from 100% to 21%, due to the lack of O_2_ source and the wanted over-oxidization to water via four-electron-transfer pathway at the open binding sites. Note that we present the data on non-modified ZnO to illustrate the typical problems of ORR at mixed O_2_ conditions and industrially relevant rates.

### Proposed solution and the characterization of ER-ZnO catalyst

**Fig. 2:**
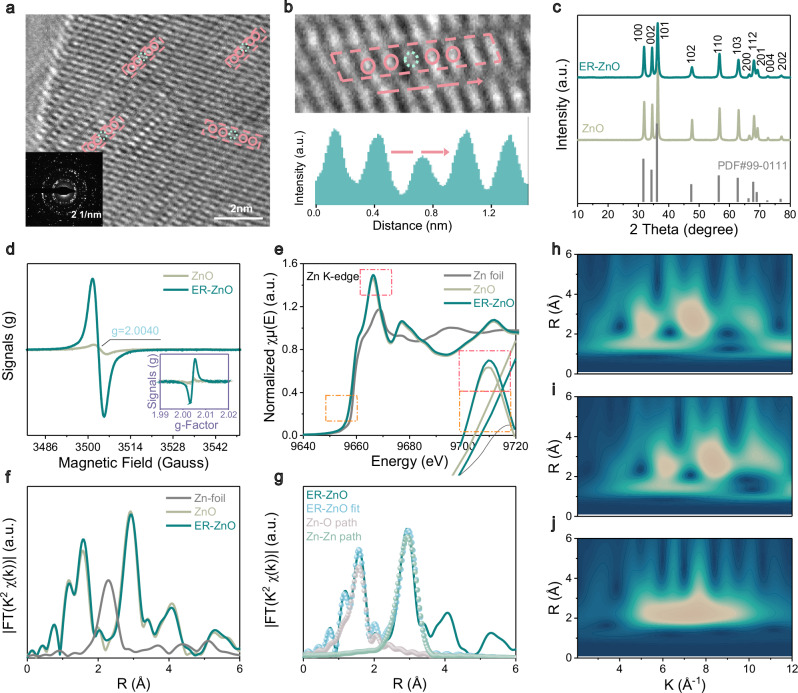
The structural characterizations of ER-ZnO catalyst. **a**, **b** High-resolution transmission electron microscopy (HR-TEM) images with typical lattice defects marked in red lines, with the intensity profile recorded for the selected area. **c** X-ray diffraction (XRD) patterns of ER-ZnO and ZnO. **d** The electron paramagnetic resonance (EPR) spectra of ER-ZnO and ZnO, with the inset as the data reproduced by g-Factor abscissa. **e** X-ray absorption near edge structure (XANES) spectra of ER-ZnO, ZnO, and Zn foils. **f** Extended X-ray absorption fine structure (EXAFS) spectra of ER-ZnO, ZnO and Zn foils. **g** Fitting curves of EXAFS spectra of ER-ZnO, ZnO and Zn foils. **h**–**j** The corresponding wavelet transform (WT)-EXAFS contour plots of ER-ZnO, ZnO and Zn foils.

Next, the ER-ZnO was synthesized via the same procedure as the reference ZnO except for adding glycerol. The glycerol was used as a modifier to synthesize zinc glycerate precursor^19^. During the calcination in air, a significant amount of O elements evaporated from zinc glycerate to form ZnO. Due to the strong interaction between Zn and O, some Zn elements also escaped from the material, resulting in structural Zn defects. The change in glycerol percentages can manipulate Zn defects in ER-ZnO. Figure 2a, b discloses the atomically dispersed zinc defects throughout ER-ZnO sample, which agrees well with the declined peak intensity at corresponding zinc-vacancy site (Fig. 2b, Supplementary Fig. 3), speaking the fact of some zinc atoms missing in the crystal structures (X-ray diffraction; Fig. 2c). The unobvious difference in XRD patterns between ER-ZnO and ZnO (i.e., only slight intensity alternations in (100), (002), and (101) crystal surfaces) provides additional evidence of the single-atom zinc vacancy nature. This conclusion is verified by other characterizations: the ER-ZnO exhibits amplified electron paramagnetic resonance (EPR) peak signal at *g* = 2.0040 relative to ZnO (4.53 vs. 0.31, Fig. 2d)^20^; the semiconductor structure of ER-ZnO also differs from the reference ZnO according to Mott-Schottky curves and electrochemical impedance spectroscopy (p-n type junction vs. n-type, Supplementary Figs. 4, 5)^21^. Quantitively, the zinc-vacancy concentration is determined to be 8.72% according to the Zn 2p/O 1*s* deconvolutions and overall survey of X-ray photoelectron spectra (XPS, Supplementary Fig. 6 and Supplementary Table 1).

The single-zinc vacancy nature of ER-ZnO is confirmed by X-ray absorption near edge structure (XANES, Fig. 2e) and extended X-ray absorption fine structure (EXAFS, Fig. 2f, g) analyses. ER-ZnO exhibits the same Zn K-edge XANES peak position as ZnO, which constitutes an upshift of 0.32 eV to high energy as compared to Zn foil (1.49 vs. 1.17 eV)^22^. This result agrees with the EXAFS of ER-ZnO and ZnO characteristic of two peaks assigning to Zn-O (1.97 Å) and Zn-Zn (3.26 Å) distances, sharply different from solely Zn-Zn distance (2.64 Å) for Zn foils (Fig. 2f, g, Supplementary Fig. 7). According to the EXAFS fitting curves (Fig. 2g–j; Supplementary Table 2), ER-ZnO has a Zn-Zn bond distance and coordination number greatly exceeding Zn foil (3.259 vs. 2.643 Å, 11.904 vs. 6.0)^23^. Interestingly, the increase in the Zn-O bond length (ER-ZnO: 1.968 Å vs. ZnO: 1.956 Å) is caused by the local deletion of Zn atoms, which migrates nearby O atoms to the defects and increases the Zn-O bond length. This is consistent with the increase in Zn–Zn bond length due to the missing of local Zn atoms (ER-ZnO: 3.259 Å vs. ZnO:3.234 Å) and decrease in the coordination number of Zn-O (ER-ZnO: 3.906 vs. ZnO: 4.006) and Zn-Zn (ER-ZnO: 11.904 vs. ZnO: 12.083) due to the unsaturated Zn sites inside the material^22,24^. Notably, our experimental results show the absence of O defects, otherwise Zn–Zn distance around the O defects would be shortened (as also confirmed by XPS elemental analysis in Supplementary Fig. 6 and EPR in Fig. 2d).

In the O K-edge XANES of ER-ZnO and ZnO (Supplementary Fig. 8), the pre-edge peaks (535.1 and 537.9 eV) are attributed to the unoccupied hybridized states of O 1*s* electrons transitioning to Zn 3*d* and O 2*p* orbitals above Fermi energy levels, splitting into two asymmetric peaks of different energies of *t*_2g_ and *e*_g_. The broad peak represents the electron transition of O 1*s* to hybridized orbitals of O 2*p* and Zn 4*sp* states, while the sharp peaks represent the electronic transitions of O 1*s* to the more localized O 2*p*_*z*_ and O 2*p*_*x+y*_ states^25^. Notably, the peak intensity of ER-ZnO (at around 535.1–537.9 eV) slightly decreases due to the reduction of available empty O 2*p* states. This suggests more charge transfer from Zn to O atoms, resulting in an increase in Zn valence state and a decrease in O valence state. These findings are consistent with Zn K-edge XANES data. Further, with the elevated oxidation state of Zn, the number of outer electrons in Zn atoms decreases, which can facilitate the bonding with electron-rich O atoms and contribute to the selective adsorption of O_2_ by ER-ZnO in the mixed N_2_/O_2_ atmosphere. At the same time, due to the decrease in outer electrons, it is difficult to provide additional electrons for bonding with O atoms, which has resulted in smaller adsorption strength of O_2_ on the ER-ZnO as compared to ZnO, endowing ER-ZnO with improved two-electron ORR activity.

We find the electronic structure closely related to electrochemical activities. ER-ZnO shows the increased Zn oxidation valence state and decreased number of outer electrons of the Zn atoms that facilitate bonding with electron-rich oxygen atoms. This can promote the selective adsorption of O_2_ by ER-ZnO in a mixed N_2_/O_2_ atmosphere. Further, due to the decrease of outer electrons, ER-ZnO cannot provide sufficient electrons for bonding with O atoms, resulting in appropriate adsorption strength of *O_2_ as compared to ZnO. According to the Sabatier’s principle^26^, the moderate adsorption strength for reactive species on ER-ZnO can demonstrate improved two-electron ORR activity^27^. These represent for us the possibility for an exciting new modulation of the atomic structure and the related electronic properties for ER-ZnO, as we can assume the presence of more electrophilic sites that allow for improved, selective O_2_ binding and thereby enhanced ORR activities.

### Quantification of performances in mixed dioxygen electroreduction

**Fig. 3:**
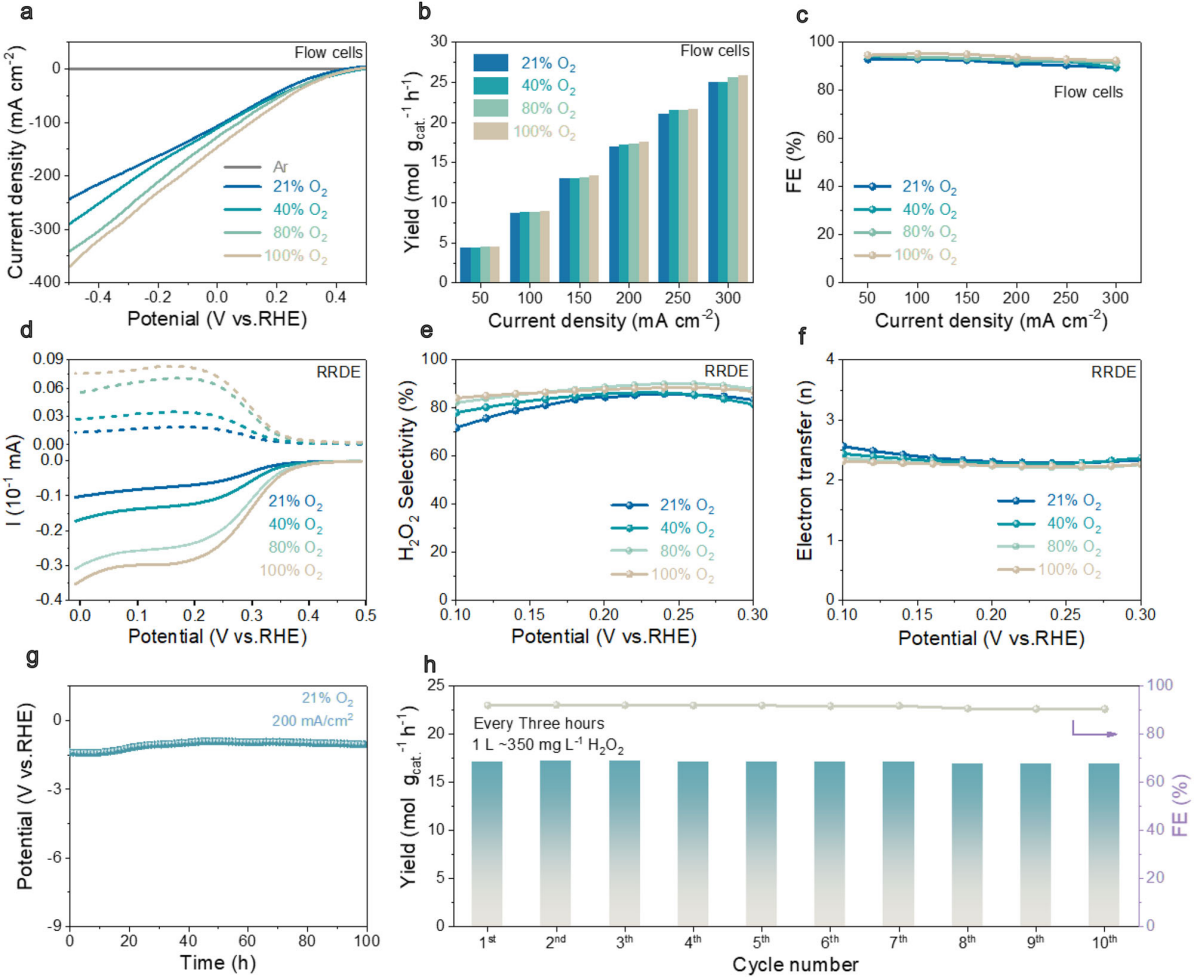
The advantages of ER-ZnO catalyst for oxygen electroreduction to H_2_O_2_ in mixed O_2_ media (21%, 40%, 80%, and 100% O_2_ in O_2_/N_2_ mixtures). **a** LSV curves tested in flow cells. **b** H_2_O_2_ yields tested in flow cells. **c** H_2_O_2_ Faradaic efficiencies tested in flow cells. **d** LSV polarization curves on RRDE at a rotation speed of 1600 rpm and scan rate of 5 mV s^−1^, where the H_2_O_2_ currents were recorded on the ring electrode at a constant potential of 1.2 V (vs. RHE). **e** H_2_O_2_ selectivity calculated on the basis of RRDE. **f** Electron transfer number (*n*) calculated on the basis of RRDE. **g** The durability test of ER-ZnO in 21% O_2_ with the current density of 200 mA cm^−2^. **h** The H_2_O_2_ yields and Faradaic efficiencies of the chronopotentiometry test under 200 mA cm^−2^ in ten times 3-h cycle tests.

Consequently, the electrocatalytic ORR performances of ER-ZnO were tested in the same condition as the reference ZnO (Supplementary Figs. 9–12). The linear sweep voltammetry (LSV) was conducted for ER-ZnO-based electrodes in Ar- and O_2_-saturated electrolytes, where the current densities in all O_2_-saturated electrolytes surpass that in Ar-counterpart indicating the occurrence of oxygen electroreduction (Fig. 3a)^28^. The ORR activities were quantified by chronoamperometric tests (Fig. 3b, c). Lowering O_2_ concentrations from 100% to 21% with the ER-ZnO catalyst, the H_2_O_2_ yields and Faradaic efficiencies show minute decay at current densities between 50 and 300 mA cm^−2^. More specifically at 300 mA cm^−2^, the H_2_O_2_ yields are 25.89, 25.60, 25.03, and 24.99 mol g_cat_^−1^ h^−1^ in 100%, 80%, 40%, and 21% O_2_, respectively. Analogously, the H_2_O_2_ Faradaic efficiencies are 92.5%, 91.5%, 89.5% to 89.3% for 100%, 80%, 40%, and 21% O_2_, respectively (Fig. 3c). Therefore, the accessibility to O_2_ is not the rate-limiting step anymore. In other words, we move the Le Chatelier reaction kinetics out of importance in the catalytic system.

The outstanding performances of ER-ZnO are validated by RRDE on drop-casting electrodes in generator-collector mode (Fig. 3d–f and Supplementary Figs. 13–16). The RRDE activities were calculated on the basis of disk current (*I*_*d*_) at the spin rate of 1600 rpm and ring current (*I*_*r*_) at the constant potential of 1.2 V. ER-ZnO, demonstrating good H_2_O_2_ molar fraction selectivity (70–90%, Fig. 3e) with the transferred electron numbers (2.0–2.6) close to that of theoretical two-electron-transfer pathway (Fig. 3f). Importantly, the ER-ZnO has demonstrated longstanding and stable H_2_O_2_ production in both mixed and high-purity O_2_ media (21% O_2_ in Fig. 3g, h and 100% O_2_ in Supplementary Fig. 17, Supplementary Table 4). Even under the industrial-level current densities, the ER-ZnO catalyst tested in 0.6 M K_2_SO_4_ has shown seldom activity degradation for 100 h at 200 mA cm^−2^. In ten-times repetitive chronoamperometric cycles, the acquired H_2_O_2_ concentration can accumulate to 350 mg L^−1^ in 1 L of 0.6 M K_2_SO_4_ every 3 h, with the Faradaic efficiencies above 90%.

### Revealing the mechanism of mixed dioxygen electroreduction

**Fig. 4:**
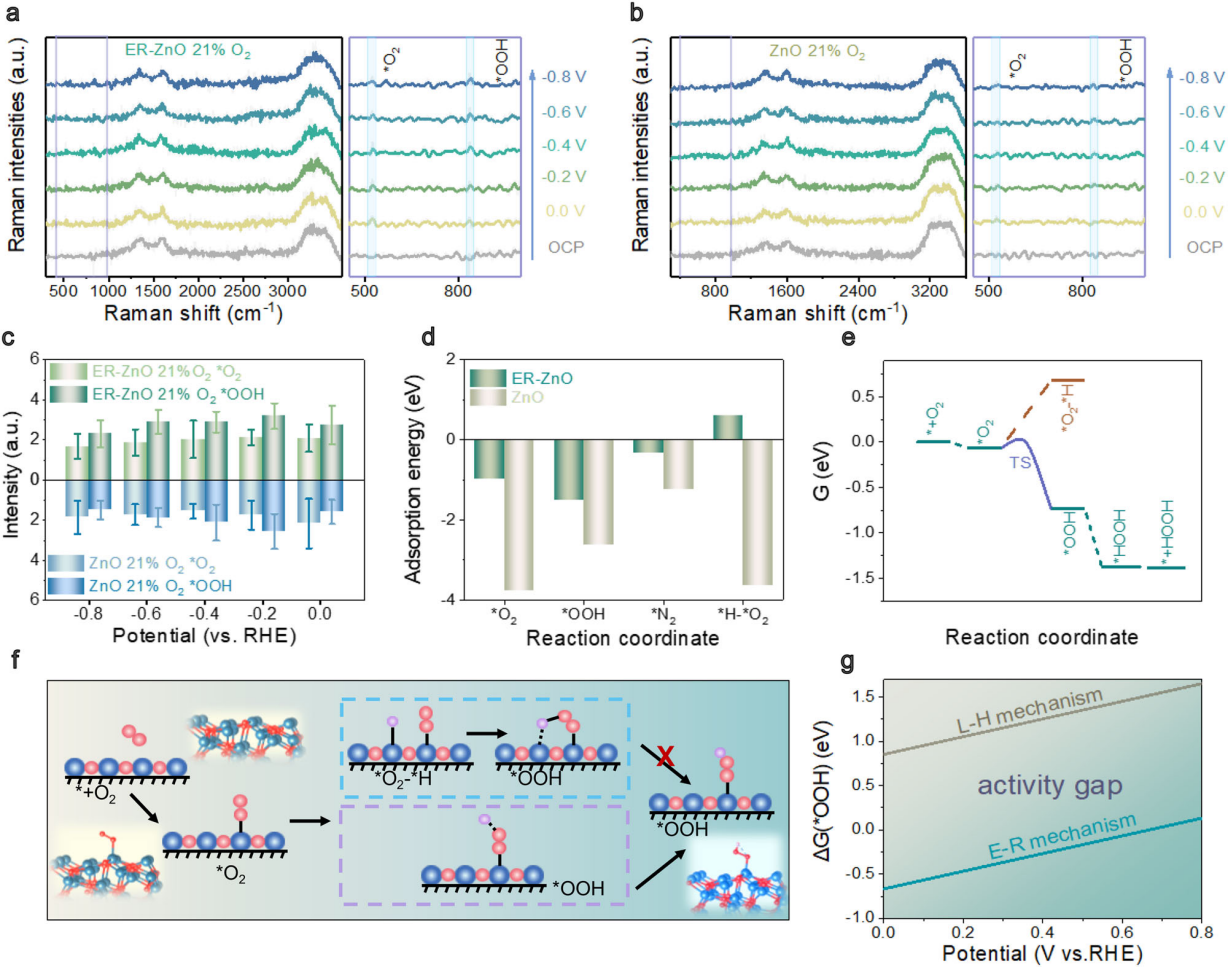
Mechanism investigation in mixed O_2_/N_2_ media (i.e., 21%O_2_—air). **a** The operando Raman spectra of ER-ZnO. **b** The operando Raman spectra of the reference ZnO. **c** The peak intensity analyses with error bar in operando Raman test. Five measurements were conducted for each data point with the error bars corresponding to the standard deviation. **d** The adsorption energy of different reaction intermediates. **e** Density functional theory (DFT) calculated free energy diagrams of ORR on ER-ZnO. **f** Reaction pathways of Eley-Rideal mechanism and Langmuir-Hinshelwood mechanism for ER-ZnO. **g** Kinetic barriers for the hydrogenation of *O_2_ to *OOH on ER-ZnO via Eley-Rideal and Langmuir-Hinshelwood mechanisms.

Motivated by the excellent ORR activities, experimental and theoretical studies are conducted to understand the catalytic mechanisms. Figure 4a, b shows the operando Raman spectra for capturing the short-lifetime ORR adsorbates (21% O_2_ in O_2_/N_2_ mixtures, Supplementary Figs. 18–20). A flow-type cell with a quartz window was developed to detect the Raman signal at 532 nm excitation wavelength from 0 to −0.8 V in 200 mV steps. The spectra of both ER-ZnO and ZnO display a subset of the following vibrational data: the band at 978 cm^−1^ originated from SO_4_^2−^ from the electrolytes^29^; the band in 1350–1600 cm^−1^ from carbon paper substrate; two potential-dependent bands at 525 and 845 cm^−1^ from O–O stretching mode of surface bound *O_2_ and *OOH, respectively^30^. The peak intensities of *O_2_ and *OOH have been analyzed in Fig. 4c. The changes in surface intermediates show the characteristic peaks for *O_2_ and *OOH in response to applied potentials. For L-H mechanism, the proton occupies part of active sites, resulting in a relatively low concentration of *O_2_ on the surface and consequently a low peak intensity (for reference ZnO). In contrast, the E-R mechanism allows for more active sites for the adsorption of O_2_, resulting in a higher *O_2_ peak intensity (for ER-ZnO). Further examination of ER-ZnO reveals the tendency of *O_2_ peak intensity decrease while *OOH increases with elevated applied potentials. This originated from E-R mechanism that causes the direct coupling between *O_2_ and dissociative H in the electrolyte, resulting in a rapid reaction rate than *OOH protonation to produce H_2_O_2_. Due to the sufficient supply of *O_2_ species in E-R mechanism, *OOH continuously accumulates on the surface of ER-ZnO, leading to the simultaneous decrease of *O_2_ species. On the other hand, the change of *OOH peak intensity shows the similar tendency to *O_2_ peak intensity on the ZnO surface. This originated from L-H mechanism, where *O_2_ binds to *H before generating *OOH, resulting in a parallel change of these two intermediates.

To experimentally probe their O_2_ selectivity, temperature programmed desorption (TPD) was conducted for the catalysts from 50 to 800 ^o^C at the heating rate of 5 ^o^C min^−1^ (Supplementary Fig. 21). The O_2_-TPD curves of both ER-ZnO and ZnO disclose three peaks at 404.9, 528.5, and 772.7 ^o^C assigning to surface adsorbed O_2_, suboxide formation and removal lattice oxygen, respectively (Supplementary Fig. 21a). The ER-ZnO has overtaken ZnO by the integrated peak area for surface adsorbed O_2_ (0.103 vs. 0.102), reflecting its high selectivity to O_2_ by providing more active centers for absorbing oxygen species. This result agrees with N_2_-TPD curves of ER-ZnO containing of two bands at 412.3 (surface adsorbed N_2_) and 528.1 ^o^C (lattice nitrogen, Supplementary Fig. 21b), demonstrating less tightly binding with N_2_ by showing smaller integrated peak areas for surface adsorbed N_2_ than ZnO counterpart (0.104 vs. 0.167). By calculating the peak area ratios of O_2_–TPD/N_2_–TPD curves, the chemical O_2_ selectivity of ER-ZnO relative to the reference ZnO is 1/0.611 (Supplementary Fig. 21c).

To elucidate the underlying origin of high O_2_ selectivity, density function theory (DFT) calculations were performed for ER-ZnO and ZnO in the mixed media (Supplementary Figs. 22–28). The density of states of Zn 3d orbital show significant occupied states near the Fermi level for both ER-ZnO and ZnO (Supplementary Fig. 29), elaborating their possible bindings with the absorbates in mixed O_2_/N_2_ electrochemical systems, including *O_2_ from O_2_, *OOH, *N_2_ from N_2_ and *O_2_-*H from the protons via the dissociation of aqueous electrolytes (Supplementary Figs. 30–33)^31^. The adsorption energy levels on ER-ZnO are −1.00 eV for *O_2_, −1.51 eV for *OOH, −0.34 eV for *N_2_ and 0.59 eV for *O_2_-*H (positive value indicative of unstable adsorption), and in accordance the reference ZnO are −3.77 eV for *O_2_, −2.62 eV for *OOH, −1.25 eV for *N_2_, and −3.63 eV for *O_2_-*H, respectively (Fig. 4d). Different from the reference ZnO favorably adsorbing all the species of O_2_, *OOH, N_2_ and protons, the ER-ZnO only binds stably with O_2_ and *OOH. Appropriate *OOH adsorption indicates it is stable on the surface, leading to high reaction activity for two-electron ORR. Further, ER-ZnO shows high selectivity to O_2_ by displaying larger binding energy relative to N_2_ (−1.00 vs. 0.34 eV), which is necessary for ORR proceeding in mixed O_2_/N_2_ atmosphere. Next, the intermediate *O_2_-*H plays a crucial role in the L-H mechanism. Upon analyzing the adsorption energies, the adsorption energy of *O_2_-*H is positive (0.59 eV), indicating the stabilization of *O_2_-*H intermediate in L-H mechanism is challenging on the surface of ER-ZnO. This is different from the negative adsorption energy on reference ZnO surface with L-H mechanism (*O_2_-*H adsorption energy: −3.63 eV). Therefore, ER-ZnO prefers E-R mechanism while ZnO prefers L-H mechanism.

The above result is consistent with the difference in charge densities (Supplementary Figs. 34, 35), which displays the favorable adsorption of ER-ZnO with *O_2_ and *N_2_ characteristic of dense accumulated electron clouds relative to *O_2_-*H^32^. Quantitively, the electron transfer numbers between ER-ZnO and different absorbates (*O_2_, *N_2_ and *O_2_-*H) have been determined by Bader charge transfer analyses (Supplementary Table 5). ER-ZnO prefers to bind with *O_2_ by transferring more electrons relative to *N_2_ (0.05 vs. 0.02e). Different from the reference ZnO characteristic of significant charge migrations after absorbing all the absorbates (0.6e for *O_2_, 0.18e for *N_2_ and 0.67e/0.42e for *O_2_/*H of *O_2_-*H), ER-ZnO could only bind with *O_2_ and *N_2_ rather than *O_2_-*H (0.05 e for *O_2_, 0.02 e for *N_2_, and −0.05e/−0.27e for *O_2_/*H of *O_2_-*H).

Overall, the reaction pathways of ORR are proposed for ER-ZnO and ZnO by using Gibbs free energy as a descriptor (Figs. 4e, f). Two-electron-transfer ORR commonly consists of four cascade steps of the catalysts adsorbing/activating dioxygen to form *O_2_, two consecutive protonations to *OOH and *HOOH, and the dissociation to H_2_O_2_ for recovering the active centers. Particularly in the potential-limiting step of *O_2_ → *OOH, two fundamental mechanisms might occur according to their proton sources (Fig. 4f, Supplementary Fig. 36): i) directly stem from bulk phase of aqueous electrolytes via E-R mechanism; ii) from the adjacent adsorbed *H via L-H mechanism. As revealed in Fig. 4e–g, the ER-ZnO prefers E-R mechanism with a free energy change of only −0.67 eV as compared to L-H mechanism (0.75 eV). This result is supported by the transition state computations of E-R mechanism for ER-ZnO by a climbing image nudged elastic band method with implicit solvation model (Supplementary Fig. 37), showing excellent reaction kinetics with the transition energy gap of only 0.057 eV (Supplementary Fig. 37). In great contrast, the reference ZnO counterpart tends to proceed via L-H mechanism rather than E-R mechanism under the same computation condition (free energy change of 0.37 vs. 1.28 eV, Supplementary Figs. 38–41).

### Prototype electrolysis device and techno-economic analysis

**Fig. 5:**
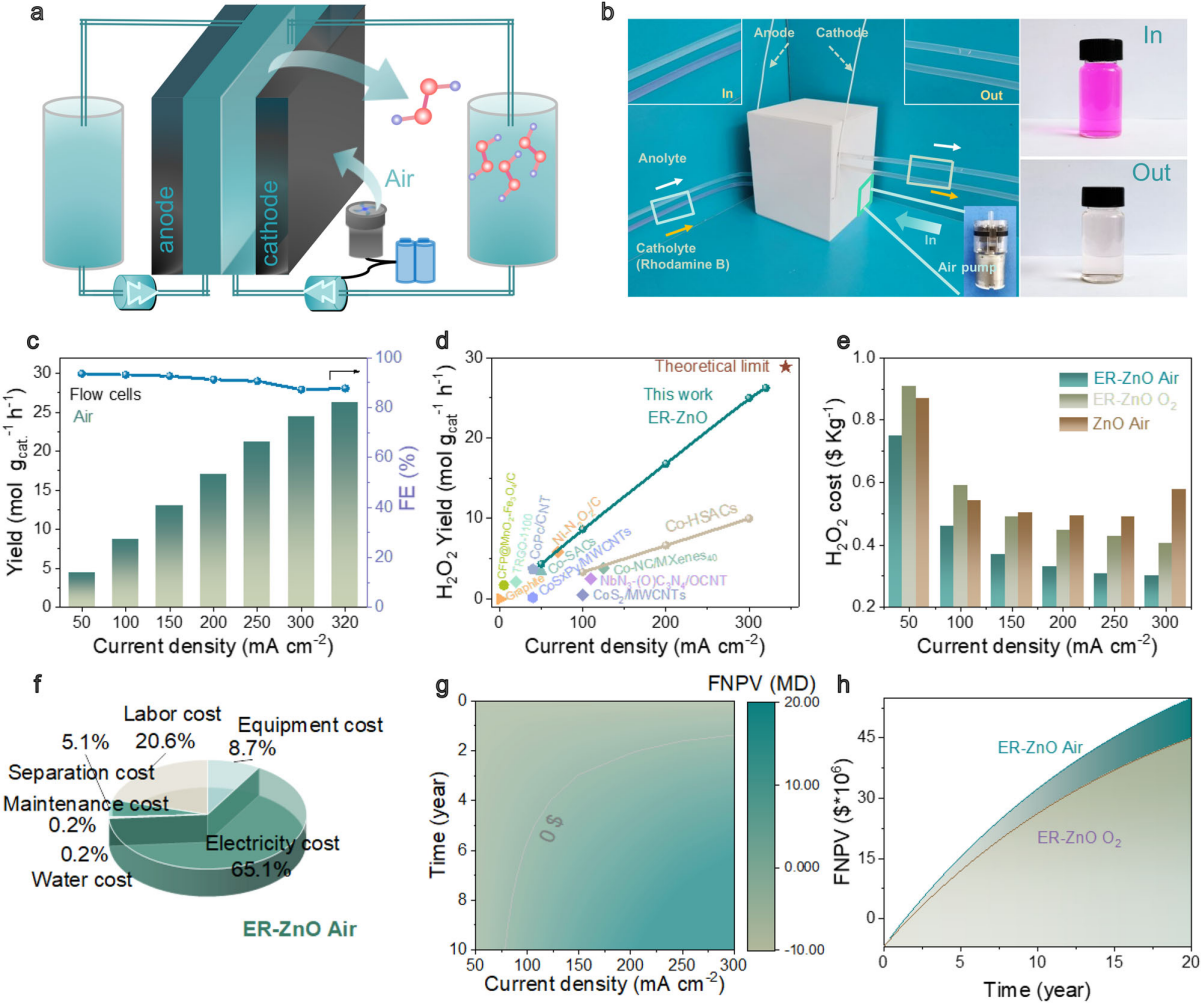
Prototype device and techno-economic analyses for oxygen electroreduction using atmospheric air feedstock. **a** The schematic diagram of the prototype device. **b** The optical picture of the prototype device operating for pollutant degradation of rhodamine B (The background color has changed from blue to green for reliable comparison). **c** The ORR H_2_O_2_ yield rates of ER-ZnO catalyst in the prototype device in atmospheric air. **d** The comparison of H_2_O_2_ yields with the state-of-the-art literature in atmospheric air, as listed in Supplementary Table 4. **e** Techno-economic analyses showing the H_2_O_2_ production cost for ER-ZnO and ZnO catalysts in atmospheric air and 100% O_2_ (pure dioxygen). **f** The proportions of H_2_O_2_ production cost for ER-ZnO in atmospheric air. **g** Financial net present value (FNPV) analyses of H_2_O_2_ production for ER-ZnO catalyst in atmospheric air. **h** Comparison of FNPV of H_2_O_2_ production for ER-ZnO in atmospheric air and 100% O_2_ (pure dioxygen).

To demonstrate the feasibility of the present model system for on-site H_2_O_2_ production, a prototype device was assembled (Fig. 5a–c), which is an integrated standalone box (size: 15 cm × 15 cm × 19 cm) composed of rechargeable battery as power sources, gas (air) pumps for flowing gases, peristaltic pumps for circulating electrolytes and flow-type cells loaded with ER-ZnO catalysts. Their ORR activities were quantified by chronoamperometric tests at different current densities (Supplementary Fig. 42). Lowering O_2_ concentrations from 100% to 21% in mixed dioxygen gas, the ORR activities of the prototype device display seldom activity decay in the current density range of 50–300 mA cm^−2^. At the current density of 300 mA cm^−2^, the prototype device demonstrates H_2_O_2_ yields (25.78, 25.94, 25.63, and 24.39 mol g_cat_^−1^ h^−1^) and Faradaic efficiencies (92.15%, 92.71%, 91.60%, and 87.17%) that comparable to above flow-type electrochemical configurations in 100%, 80%, 40%, and 21% O_2_, respectively. Even in much lower O_2_ concentrations (i.e., 5%, 10% and 15% O_2_; Supplementary Figs. 43–45), the prototype device can still show superior activities approaching theoretical limits (Supplementary Fig. 46).

Next, the prototype device has operated directly using atmospheric air feedstock, displaying the H_2_O_2_ yields and Faradaic efficiencies (such as 24.41 mol g_cat_^−1^ h^−1^ and 89.24% at 300 mA cm^−2^) comparable to those in mixed dioxygen media. This result is consistent to RRDE test showing analogous results to mixed dioxygen (Supplementary Fig. 47). By elevating the current density to 320 mA cm^−2^, the prototype device has demonstrated the highest H_2_O_2_ yield and Faradaic efficiency (26.2 mol g_cat_^−1^ h^−1^ and 87.76%; Fig. 5c and Supplementary Fig. 48), approaching the theoretical limit for direct air electrolysis (~345.8 mA cm^−2^). To our best knowledge, the ORR activity of ER-ZnO rates as the best in the literature for direct air electrolysis (Fig. 5d and Supplementary Table 6).

Consequently, the practical application of the prototype device was demonstrated for the in situ oxidative degradation of a model dye used for referencing degradation experiments^33^. To evaluate the degradation ability, an aqueous solution was made of rhodamine B in K_2_SO_4_ aqueous electrolyte. The solution flowed through the prototype device operating in atmospheric air at 300 mA cm^−2^, where the color of rhodamine B solution fades rapidly. The optical images of electrolyte (Fig. 5b) and Supplementary video clearly displayed the dye decolorization after the prototype device operating in the established condition.

Finally, techno-economic analyses have been conducted under scalable and industrially viable conditions^33,34^. The H_2_O_2_ production cost from ORR contains of capital investment and operating cost, linking to more detailed system set-ups of energy (electricity), feedstocks (mixed O_2_ source and water), electrolysis cell, labor cost, maintenance cost, etc. Different from many previous reports of roughly calculating the average H_2_O_2_ cost from only energy and feedstock input, here a rigorous simulation model has been built by taking account of all of the possible parameters (Supplementary Tables 7–9). The accuracy of the models was examined by comparing the simulation results of ER-ZnO at 300 mA cm^−2^ with that from manual calculations according to Faraday law, and both of the data are consistent (please see experimental section for details).

The cost breakdown for H_2_O_2_ is calculated by normalizing both capital investment and operating cost, and presented as a function of current densities (Fig. 5e). In the mixed O_2_ condition (i.e., 21% O_2_ or air), the H_2_O_2_ production cost of ER-ZnO decreases with the elevated current densities, from $0.75 kg^−1^ at the current density of 50 mA cm^−2^ sharply down to $0.33 kg^−1^ at 200 A cm^−2^, and slowly to the lowest price of $0.30 kg^−1^ at 300 mA cm^−2^. While under the same test condition in pure dioxygen (i.e., 100% O_2_), the minimal H_2_O_2_ production costs for ER-ZnO are 0.40 $ kg^−1^ at 300 mA cm^−2^, indicating the merit of mixed O_2_ sources for economic manufacture. Notably, the H_2_O_2_ production costs of ER-ZnO ($0.30 kg^−1^) only account 20% of market prices from traditional anthraquinone oxidation/reduction process ($1.5 kg^−1^).

To examine the contributions of different parameters to H_2_O_2_ production cost, single variable sensitivity has been analyzed for ER-ZnO (Fig. 5f, Supplementary Fig. 49 and Tables 10, 11). In the mixed O_2_ condition (21% O_2_-air), the H_2_O_2_ production cost is most susceptible to the variation in electricity price (65.1%), followed by human labor (20.6%), equipment (8.7%), separation (5.1%), maintenance (0.2%) and water cost (0.2%). Therefore, cheap electricity is critical to achieving low production cost, that is, with each US$0.01 kWh^−1^ corresponding to a H_2_O_2_ price change of US$0.002 kg^−1^ (Fig. 5e). This result is different from the H_2_O_2_ production cost of ER-ZnO in pure dioxygen (100% O_2_, Supplementary Fig. 49), showing it susceptible to the variation in both electricity (51.3.1%) and O_2_ (24.2%) prices, followed by human labor (14.5%), equipment (6.1%), separation (3.6%), water cost (0.2%) and maintenance (0.1%). Each US$0.01 kWh^−1^ of O_2_ corresponds to a H_2_O_2_ price change of US$0.001 kg^−1^ (Supplementary Fig. 49).

The accumulative economic profit has been evaluated by financial net present value (FNPV) according to the following equation:

3 $${{{{\rm{FNPV}}}}}={\sum }_{t=0}^{n}{(Cl-CO)}_{t}\times {(1+i)}^{-t}$$

where Cl is the present value of future cash flow (H_2_O_2_ product revenue), *CO* is the present value of the original investment (capital cost, operating cost and 25% tax), *i* and *t* are the discount rates and the duration (payback time), respectively. The FNPV and payback time for ER-ZnO in mixed O_2_ media (21% O_2_ or air) are illustrated according to the current densities (Fig. 5g and Supplementary Fig. 42). At the operating current density of 50 mA cm^−2^, the FNPV for ER-ZnO starts at $−6,948,946 and ends at $−5,107,495 through the whole lifespan. The negative FNPV values indicate a non-profit manufacture. Notably, the payback duration is reduced by elevating current densities, for example, 6 years for operating at 100 mA cm^−2^ (FNPV value: $8,606,199) and 2 years for operating at 300 A cm^−2^ ($54,815,167), underlining the importance of high reaction rates for economic manufacture. Besides current densities, the O_2_ source is underlined as an important evaluation parameter, because ER-ZnO in pure dioxygen (100% O_2_) only arrives at $45,048,833 at 300 mA cm^−2^ under the same condition (Fig. 5h and Supplementary Tables 10, 11). The use of a mixed O_2_ source (21% O_2_ or air) could bring 21.7% better economics for ER-ZnO as comparison to that in purity dioxygen (100% O_2_). Collectively, the techno-economic analyses indicate our model is a promising candidate for industrial H_2_O_2_ synthesis.

## Discussion

The recent studies show tremendous examples of how to harness chemical/catalytic systems by leveraging specific reaction parameters (such as concentrations^35^, pressures^36^ and temperatures^37^_)_, presumably under the assumption of the basic rule of Le Chatelier principle applying. In this work, we have developed a single-atom vacancy model for the oxygen electroreduction to H_2_O_2_, which apparently bypasses the macroscopic Le Chatelier’s kinetics. We show the prominent ORR activities in a wide range of O_2_ levels. Further techno-economic analyses demonstrate the advantages of oxygen electroreduction in mixed dioxygen condition as comparison to high-purity O_2_ and conventional anthraquinone process. This work would have general implications, giving insight into the rational design and tailoring of heterogeneous catalytic systems “beyond” the constraint of classical Le Chatelier principle, approaching the substrate binding behavior typical for natural enzymes.

## Methods

### Material synthesis

ER-ZnO was synthesized by dissolving Zn(OAc)_2_ (0.26 g) in a mixed solution containing of ethanol (30 mL) and glycerol (2 mL). Next, the mixture was transferred into Teflon-lined stainless-steel autoclave for hydrothermal reaction at 180 °C for 24 h. The as-obtained solid precursor (i.e., zinc glycerolate) was collected and thermally annealed at 400 °C for 1 h. Other comparison samples, such as ZnO and ER-ZnO-X (*X* = 1, 3 and 4), were synthesized via a similar procedure without glycerol or with different amounts of glycerol (i.e., 1, 3, and 4 mL, respectively).

### Rotating ring disk electrode (RRDE) system

Pt wire and Ag/AgCl electrodes were used as counter and reference electrodes, respectively. RRDE (PINE Research Instrument) was employed as working electrode. The catalyst ink was prepared by dip-coating method as follows: 5.0 mg of catalyst powder, 1.0 mg of carbon black and 30 μL of Nafion solution were dispersed in 970 μL of isopropanol. After bath sonication for 30 min, 10 μL of the as-obtained catalyst ink was dropped onto the surface of disk electrode of RRDE followed by dry under ambient condition (catalyst loading: 0.2 mg cm^−2^).

All of the ORR data were measured in 0.6 M K_2_SO_4_ aqueous solution. Prior to the test, cycle voltammetry measurement was applied on RRDE at the scan rate of 50 mV s^−1^ until reaching a stable state. Then the ORR polarization curves were obtained by LSV with a sweep rate of 10 mV s^−1^ at 1600 rpm. To detect the as-generated H_2_O_2_, the potential of 1.2 V (vs. RHE) was applied to the Pt ring electrode. The H_2_O_2_ selectivity and electron transfer number (*n*) were calculated on the basis of disk current (*I*_*d*_) and ring current (*I*_*r*_) according to the following equations:

4 $${H}_{2}{O}_{2}\,selectivity\,(\%)=200\frac{{I}_{r}/{N}_{c}}{|{I}_{d}|\,+\,{I}_{r}/{N}_{c}}$$

5 $$n=4\frac{|{I}_{d}|}{|{I}_{d}|+\,{I}_{r}/{N}_{c}}$$

### Flow-type electrochemical cells

The flow-type cell was built by anodic and cathodic compartments separated by Nafion 115 membrane. The ER-ZnO catalyst was loaded on the GDE (working area: 1 cm^2^) with a mass loading of 0.2 mg cm^−2^. Next, the GDE attached with Teflon film was used as the cathode. IrO_2_-coating titanium sheet and Ag/AgCl electrode were employed as the anode and reference electrode, respectively. Particularly for cathodic and anodic compartments, 50 mL 0.6 M K_2_SO_4_ aqueous solution was used as the electrolyte and recycled with the peristaltic pump at the rate of 40 r min^−1^. The H_2_O_2_ concentration was measured after operatingg for 5 mins. The gas supply rate was stabilized at 20 mL min^−1^ feeding into the cathodic compartment.

### Determination of H_2_O_2_ product

The H_2_O_2_ concentration was detected by using UV–vis spectra with TiOSO_4_ chromogenic reagent. Specifically, 9 mL of cathodic electrolyte was mixed with 1 mL of TiOSO_4_ chromogenic reagent composed of titanium (IV) sulfate (14 mg) and concentrated sulfuric acid (0.2 mL). The diluted sulfuric acid can hydrolyze titanium sulfate into titanium oxysulfate (TiOSO_4_), and react with H_2_O_2_ in the cathodic electrolyte to form a yellow complex according to the following equations:

6 $$Ti{(S{O}_{4})}_{2}+{H}_{2}O=TiOS{O}_{4}+{H}_{2}S{O}_{4}$$

7 $$TiOS{O}_{4}+{H}_{2}{O}_{2}=[TiO({H}_{2}{O}_{2})]S{O}_{4}(Yellow\;complex)$$

Subsequently, the amount of H_2_O_2_ was detected by analyzing the homogeneous mixed solution through UV–vis spectroscopy at the wavelength of 408 nm (supplementary Fig. 2).

The H_2_O_2_ yield (mol g^−1^_cat_ h^−1^) and Faradaic efficiency (FE) for the H_2_O_2_ production were calculated according to the equation:

8 $$Yield=\frac{{C}_{{H}_{2}{O}_{2}}{V}_{electrolyte}}{t\,{m}_{cat}}$$

9 $$FE\,(\%)=\frac{2F{C}_{{H}_{2}{O}_{2}}{V}_{electrolyte}}{34It}\times 100\%$$

Where ${C}_{{H}_{2}{O}_{2}}$ is H_2_O_2_ concentration (mol L^−1^), ${V}_{electrolyte}$ is the volume of cathodic electrolyte (*L*), *F* is the Faraday constant (96485 C mol^−1^), 34 is the molar mass of H_2_O_2_, *t* is reaction duration, *I* is the applied steady current and ${m}_{cat}$ is catalyst mass loading (mg cm^−2^).

### Operando Raman spectroscopy

Operando Raman spectra were collected on a high-resolution Raman spectrometer equipped with external optical path and CHI1140C electrochemical workstation. Firstly, a catalyst ink was prepared by mixing 10 mg of catalyst powder and 60 μL of Nafion in 1.0 mL of ultrapure water, which was then sprayed on a carbon paper (2.5 cm × 2.5 cm). The catalyst-coated carbon paper, Pt foil and Ag/AgCl electrode were employed as working, counter and reference electrodes, respectively. A flow-type cell with transparent module was designed to assemble above electrodes and construct three-phase interfaces for exposing to laser light. All of the detected spectra were collected with 532 nm laser wavenumber in the same working environment.

### Prototype device assembly

The device was assembled into a standalone box (size: 15 cm × 15 cm × 19 cm) composed of battery power, gas pump, two peristaltic pumps, electrolysis cell and two flasks for holding electrolytes. The electrocatalytic performances of this prototype device have been tested in natural air. To explore the potential utilization for degradation experiment, rhodamine B (Concentration: 0.03 mg mL^−1^) was added into cathodic electrolyte during the operation of prototype device.

## Supplementary information

Supplementary Information

Peer Review File

Description of Additional Supplementary Information

Supplementary video

## Data Availability

The datasets generated and analyzed during the present study are included in the paper and supplementary information. Source data are provided upon request.
