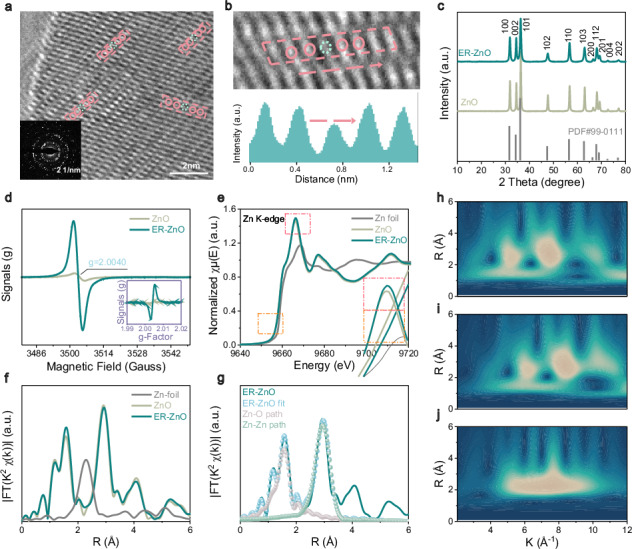# Author Correction: Single-zinc vacancy unlocks high-rate H_2_O_2_ electrosynthesis from mixed dioxygen beyond Le Chatelier principle

**DOI:** 10.1038/s41467-025-56841-7

**Published:** 2025-02-11

**Authors:** Qi Huang, Baokai Xia, Ming Li, Hongxin Guan, Markus Antonietti, Sheng Chen

**Affiliations:** 1 Key Laboratory for Soft Chemistry and Functional Materials, School of Chemistry and Chemical Engineering, Nanjing University of Science and Technology, Ministry of Education, Nanjing, 210094 China; 2 Max Planck Institute of Colloids and Interfaces, Potsdam, 214476 Germany

**Keywords:** Electrocatalysis, Electrocatalysis, Electrocatalysis

Correction to: *Nature Communications* 10.1038/s41467-024-48256-7, published online 16 May 2024

In the version of the article initially published, Fig. 2i contained an incorrect wavelet transform (WT)-EXAFS contour plot for comparison sample (ZnO), as seen in Fig. 1. This figure has now been updated in both the PDF and HTML versions of the article.

Original Figure:
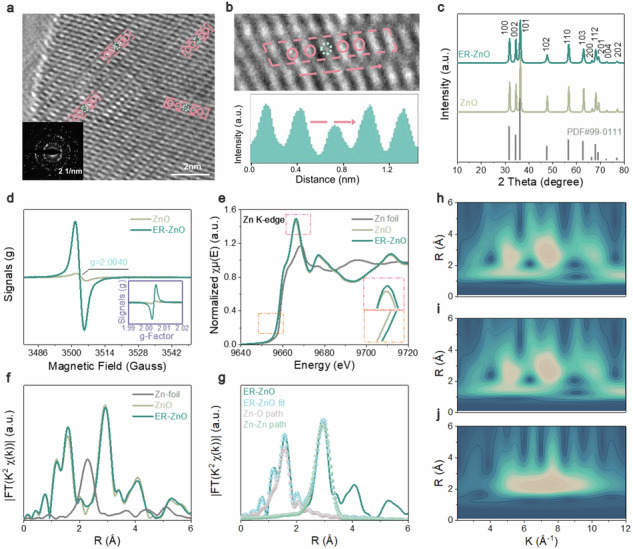


Corrected Figure: