# Supplementary material for: Single-zinc vacancy unlocks high-rate H2O2 electrosynthesis from mixed dioxygen beyond Le Chatelier principle
Source: Nat Commun. 2024 May 16;15:4157. doi: 10.1038/s41467-024-48256-7 (PMC11098813; doi:10.1038/s41467-024-48256-7)
Supplement: Supplementary file 1 — Supplementary Information [file 41467_2024_48256_MOESM1_ESM.pdf]

Supplementary Information

Single-zinc vacancy unlocks high-rate H_2O_2 electrosynthesis from mixed dioxygen beyond Le Chatelier principle

Qi Huang[1], Baokai Xia[1], Ming Li[1], Hongxin Guan[1], Markus Antonietti,[2] Sheng Chen[1,2*]

[1] Key Laboratory for Soft Chemistry and Functional Materials, School of Chemistry and Chemical Engineering, Nanjing University of Science and Technology, Ministry of Education, Nanjing, 210094, China

[2] Max Planck Institute of Colloids and Interfaces, Potsdam, 214476, Germany

E-mail: sheng.chen@njust.edu.cn

1. Supplementary Methods

(1) Materials characterization.

Transmission electron microscopy (TEM) was conducted on a FEI talos F200x G2 TEM. X-ray diffraction (XRD) was conducted on a SmartLab SE with Cu Kα radiation operating at 40 kV and 30 mA. X-ray photoelectron spectroscopy (XPS) was collected on a Thermo Scientific K-Alpha with Al Kα X-ray source. Electron Paramagnetic Resonance (EPR) was conducted on a Bruker EMXplus-6/1. Fourier transform infrared (FT-IR) was conducted on a Nicolet iS20 FT-IR spectrometer. Zn K-edge X-ray absorption near edge structure (XANES) and extend X-ray absorption fine structure (EXAFS) experiments was conducted at shanghai Synchrotron Radiation Facility.

(2) Computational methods

DFT calculations were performed by the MedeA-Vienna Ab initio Simulation Package (VASP). The Perdew-Burke-Ernzerhof (PBE) generalized gradient approach was used to define the exchange-correlation potential.[1, 2] The interaction between the atomic cores and electrons was described by using the projector augmented wave method (PAW).[3, 4] The plane wave energy cutoff was set to be 400 eV. The Brillouin zone in the real space was sampled with a 1 × 1 × 1 Monkhorst-Pack K-point grid. The convergence criterion was set to be 10^{-5} eV and 0.02 eV/Å for energy and force in the geometry optimizations, respectively. A Gaussian smearing method was employed with 0.05 eV width. Hubbard-U correction method (DFT+U) was carried out to improve the description of highly correlated Zn 3d orbitals with the value of U-J set to be 2.5 eV.

The detailed Gibbs free energy has been calculated according to the following equation:

$$G = E + ZTE - TS \tag{1}$$

Where G, E and ZTE refer to chemical Gibbs free energy, electronic energy and zero-point energy, respectively. The entropy can be calculated by the sum of the vibrational, rotational, translational, and electronic contribution as to:

$$S=S_v+S_r+S_t+S_e \tag{2}$$

Since $S_e \approx 0$ at the fundamental electronic level.

For the case of solids and adsorbates, some approximations can be adopted: Translational and rotational motions can be omitted, therefore, $S_t \approx 0$ and $S_r \approx 0$. In this case, all the entropy values come from the vibrational contribution: $S = S_v$

Finally, Gibbs free energy for different states was calculated as to:

$$G = E + ZTE - TS_v \tag{3}$$

Further, climbing image Nudged Elastic Band (Cl-NEB) method with implicit solvation model was employed to compute kinetic barrier for the transition states. Six images were interpolated between the initial (IS) and the final state (FS) to determine minimum energy path (MEP), and geometry of the transition state (TS). The total energy and force thresholds for geometry optimizations were 1×10^{-5} eV and 0.5 eV/Å, respectively. Transition states were confirmed through frequency analysis to ensure imaginary frequency existed, assigned to MEP's unstable mode.

(3) Technoeconomic analysis

3.1 Process simulation

The process simulations were realized by softwares, for example, DWSIM, COCO and MATLAB. Important parameters were input into these softwares, and the simulation results are compared, analyzed, and optimized to carry out practical applications.[5, 6]

3.1.1 Physical property selection and input data of main logistics

The electrolyte used in the whole process system is K_2SO_4, forming an aqueous electrolyte

solution.[7-9] The input of streams is shown in Supplementary Table 5. Considering the running state of the whole flow stock, it is set as 8000kg/hr. Since the utilization rate of gas cannot exceed 25%, the circulation amount of gas is set as 15000kg/hr.

3.1.2 Electrolysis device

Electrolysis device is the core equipment of whole process flow, which is mainly composed of cathodic and anodic electrodes, separation membranes, electrolytes and other components. The device has four material inlets: cathode, anode, reaction gas and electrical power.[10]

The possible reactions are listed as follows:

$$\text{Anodic part: } H_2O - 4e^- \rightarrow 4H^+ + O_2 \tag{4}$$

$$\text{Cathodic part: } O_2 + 2H^+ + 2e^- \rightarrow H_2O_2 \tag{5}$$

$$\text{Overall: } O_2 + 4H^+ + 4e^- \rightarrow 2H_2O \tag{6}$$

According to the experiment, the side reaction is four-electron-transfer ORR process to produce H_2O. The change of heat is also considered due to the enthalpy difference between products and reactants in the reaction process of the electrolytic cells.[11, 12]

3.1.3 The design of the whole simulation process

<u>Determine the type of electrolytic cell</u>

According to the actual production demand, the reaction equation included in the device, the working mode, and the structural characteristics of the electrolytic cell have been determined.[13]

<u>Determine the scale of production</u>

The reaction area of the electrolysis cell is 1000 m^2.

<u>Calculation of reaction current</u>

$$I = I_S \times s_{rea} \tag{7}$$

Where I is the total current, I_s is the current density and s_{rea} is the reaction area.

Calculate the production of H_2O_2

In the process of unit electrolysis, the key substances are O_2, H_2O_2, and H_2O. The Faraday law is used for calculation:

$$m = \frac{I \times M \times t \times FE}{n \times F} \tag{8}$$

Where *m* represents the mass of H_2O_2 (g), *M* is the molecular mass of H_2O_2, *Q* is the electricity quantity (C), *I* is current (A), *t* is time (s), and *n* is the electron transfer number of the reaction.

3.2 Economic analyses

3.2.1 Verify the accuracy of the simulations

The accuracy of simulated output results is verified through calculations. Specifically, For ER-ZnO in the prototype device, the ORR current density is 300 mA cm^{-2}, and Faradic efficiency (FE) of 87.2%. The concentration of the H_2O_2 product is set as 70 wt%.

Therefore, the total current is:

$$I = I_S \times s_{rea} = 0.3\frac{A}{cm^2} \times 1000\ m^2 \times \frac{10000\ cm^2}{m^2} = 3000000\ A \tag{9}$$

The power needed is given by:

$$power = UI = 3.22\ V \times 3000000\ A = 9660000\ W = 9660\ kW \tag{10}$$

The yield rate of 70wt% H_2O_2 is:

$$\frac{m}{hr} = \frac{I \times M \times t \times FE}{n \times F \times 1\ hr} = \frac{3000000\ A \times 34\frac{g}{mol} \times 3600\ s \times 87.2\%}{2 \times 96485\frac{C}{mol} \times 70\% \times 1\ hr} = 2370452.846\frac{g}{h}$$
$$= 2370.453\ kg/hr \tag{11}$$

It is assumed that the byproduct is H_2O ($O_2 + 4H^+ + 4e^- \rightarrow 2H_2O$), so the flow rate is:

$$\text{byproduct}(H_2O) = \frac{I \times M \times t \times FE}{n \times F \times 1\ \text{hr}} = \frac{3000000\ \text{A} \times 18 \frac{\text{g}}{\text{mol}} \times 3600\ \text{s} \times 12.8\%}{4 \times 96485 \frac{\text{C}}{\text{mol}} \times 1\ \text{hr}} \times 2$$
$$= 128948.541 \frac{\text{g}}{\text{hr}} = 128.949\ \text{kg/hr} \tag{12}$$

And the cathode needs to separate 2047.868 kg/hr 70wt% H_2O_2, that is, the amount of H_2O required is:

$$\text{The cathode theory requires } H_2O = 2370.453 \frac{\text{kg}}{\text{hr}} \times (1 - 70\%) = 711.136\ \text{kg/hr} \tag{13}$$

Because the byproduct is H_2O, the actual need to replenish water is:

$$\text{The actual need to replenish } H_2O = 711.136 \frac{\text{kg}}{\text{hr}} - 128.949 \frac{\text{kg}}{\text{hr}} = 582.186\ \text{kg/hr} \tag{14}$$

Because both the main and the side reactions consume O_2, the theoretical value of O_2 required per hour is:

$$\text{The consumption of oxygen} = \frac{m_{H_2O_2} \times M_{O_2}}{M_{H_2O_2}} \times 1 + \frac{m_{H_2O} \times M_{O_2}}{M_{H_2O}} \times \frac{1}{2}$$
$$= \frac{2370.453\ \text{kg/hr} \times 0.7 \times 32\ \text{g/mol}}{34\ \text{g/mol}} \times 1$$
$$+ \frac{128.949\ \text{kg/hr} \times 32\ \text{g/mol}}{18\ \text{g/mol}} \times \frac{1}{2} = 1676.332\ \text{kg/hr} \tag{15}$$

So the H_2O consumption for the anodic OER reaction is:

$$\text{Anode}(H_2O) = \frac{I \times M \times t \times 2}{n \times F} = \frac{3000000\ \text{A} \times 18\ \text{g/mol} \times 3600\ \text{s} \times 2}{4 \times 96485\ \text{g/mol} \times 1\ \text{hr}}$$
$$= 1007410.478\ \text{g/hr} = 1007.410\ \text{kg/hr} \tag{16}$$

The O_2 for the anodic OER reaction is:

$$\text{Anode}(O_2) = \frac{I \times M \times t}{n \times F \times 1\ \text{hr}} = \frac{3000000\ \ \text{A} \times 32\ \text{g/mol} \times 3600\ \text{s}}{4 \times 96485\ \text{g/mol} \times 1\ \text{hr}} = 895475.981 \frac{\text{g}}{\text{hr}}$$
$$= 895.476\ \text{kg/hr} \tag{17}$$

The above results are comparable to those calculated by software, so it verifies the accuracy of our software simulations. Some minor discrepancies are owning to the following reasons: i) the heat

is significantly increased because of enthalpy difference between different materials; ii) O_2 leaves with water vapor, resulting in a more H_2O consumption than the theoretical value. Please see specific simulation data in the following Supplementary Tables 7-9.

3.2.2 Capital Cost calculation

From the DOE H_2A analysis for central grid electrolysis, the electrolyzer cost for the stack component is \$1619 m^{-2}. The instillation factor is 1.2. Thus, the cost for the reference electrolyzer is:

$$\text{Electrolyzer cost} = 1000\ \text{m}^2 \times \frac{\$1619}{\text{m}^2} \times 1.2 = \$1942800 \tag{18}$$

From the H_2A, the balance of plant capital cost is 35% of the total cost, while the stack is 65%:

$$\text{Bop capital cost} = \$1942800 \times \frac{0.35}{0.65} = \$1046123.077 \tag{19}$$

The capital cost of membrane separation is estimated based on distillation.

$$\text{Membrane separation cost} = \$10664000 \times (\frac{159.234\ \text{L/min}}{1000\ \text{L/min}})^{0.7} \times 0.7$$
$$= \$2062734.06 \tag{20}$$

We depreciate the cost of fixed capital to each year with a period of 20-years, and the interest rate is 3.25%.

$$\text{Depreciation of fixed capital}$$
$$= \frac{(\$1942800 + \$1046123.077 + \$2062734.06) \times 3.25\%}{1 - \frac{1}{(1+3.25\%)^{20}}}$$
$$= \$347447.340/\text{year} \tag{21}$$

3.2.3 Operating Costs

The device work for 8000 hours per year, and the rest for equipment maintenance. We then simulate the energy consumption of different sections, including heat exchangers, pumps and compressors. The operation cost is thus calculated.[14, 15] The running results are shown in Supplementary Tables 8-9.

Assuming the electricity price is 3 cents/kWh, the electricity cost is calculated according to the electricity demand and price:

$$\text{Electricity cost} = 10870.948\ \text{kW} \times \frac{\$0.03}{\text{kWh}} \times 8000\frac{\text{h}}{\text{year}} = \$2609027.520/\text{year} \tag{22}$$

The maintenance cost is assumed as 2.5% of capital cost per year (from H_2A):

$$\text{Maintenance cost} = \$347447.340/\text{year} \times 2.5\% = \$8686.183/\text{year} \tag{23}$$

Notably, the cost of air is $0. The industrial O_2 price is $0.1/kg (source: https://www.intratec.us/chemical-markets/oxygen-price).

The price of industrial water is $0.00191/gallon or $0.0005046/kg (https://www.fbgtx.org/673/Industrial-Water-Rates). The cost of water is:

$$\text{H}_2\text{O cost} = 1783\frac{\text{kg}}{\text{hr}} \times \frac{\$0.0005046}{\text{kg}} \times 8000\frac{\text{hr}}{\text{year}} = \$7197.614\ /\text{year} \tag{24}$$

The operating cost of membrane separation is:

$$\text{Separation cost} = \$2062734.06 \times 10\%\ /\text{year} = \$206273.406\ /\text{year} \tag{25}$$

According to the average wage of the American manufacturing industry, it is estimated that the labor cost in the first year will be $500,000, and the annual wage will increase by 5%. Therefore, the total labor cost for 20 years is $16532977.05.

The cost price of H_2O_2 is:

The cost price of H_2O_2

$$= \frac{\left(\frac{\$347447.340}{\text{year}} + \frac{\$2609027.520}{\text{year}} + \frac{\$8686.183}{\text{year}} + \frac{\$7197.614}{\text{year}} + \frac{\$206273.406}{\text{year}}\right) \times 20\ \text{year} + \$16532977.05}{\frac{2368\text{kg}}{\text{h}} \times 70\% \times \frac{8000\text{h}}{\text{year}} \times 20\ \text{year}}$$

$$= \$0.302\ \text{kg}^{-1} \tag{26}$$

The price is given by the product revenue minus operating costs, and 25% tax. The market price of 70 wt% H_2O_2 is reported as \$0.556/kg.

In addition, The FNPV is analyzed, indicating that the scheme is feasible and the investment benefit is excellent (Supplementary Tables 10, 11).

$$\text{FNPV} = \sum_{t=0}^{n} (\text{Cl} - \text{CO})_t \times (1 + i)^{-t} \tag{27}$$

Where Cl is the present value of future cash flow, CO is the present value of the original investment, i is the discount rate, and t is the duration.

In addition, our on-site power generation method does not require transportation and storage. In contrast, the H_2O_2 cost of the traditional industrial anthraquinone production process without transportation and storage cost is about \$1.5/kg (http://www.h2o2.com/faqs/FaqDetail.aspx?fId=25). Therefore, we can conclude that our strategy is very competitive when compared to the current anthraquinone method for H_2O_2 production.

2. Supplementary Notes

Supplementary Note 1:

According to the literature,[16-20] the advantages and disadvantages of as-proposed single-zinc vacancy catalyst have been discussed:

i) Advantages: firstly, the raw materials for synthesizing our single-zinc vacancy catalysts are easily obtainable (glycol and zinc acetate), and the synthesis conditions are mild, which has been achieved by the calcination at low temperature in air. In contrast, traditional single-atom catalysts typically require complex and severe synthetic conditions, for example, calcination at high temperature or etching in strong acids. Secondly, single-zinc vacancy catalysts have high stability and numerous active sites. The metal defects on the surface of metal oxides do not result in a significant decline of active sites or structural failure. In contrast, traditional single-atom catalysts consist of atomically dispersed metal sites with a low content but high work function. During the reaction process, single metal atoms would tend to peel off or agglomerate, leading to the decline of active sites and consequently catalytic activities.

ii) Disadvantages: single-zinc vacancy catalyst has low atom utilization due to the fact of only metal sites on the outer surface contributing to catalyzing the reactions, while those zinc atoms in the bulk phase have not been directly involved in the reaction. In contrast, traditional single-atom catalysts are typically supported on low-dimensional materials with high surface area. This allows for highly exposed surface atoms for contacting with the reactants, leading to high atom utilizations.

Supplementary Note 2:

The onset potential changes of ZnO are 0.036 and 0.035 V in flow cell and RRDE systems from 100% O_2 to 21% O_2, respectively (Supplementary Table 3). This is in compliance with the Le Chatelier Principle. While under the same condition, the onset potential changes of ER-ZnO are only 0.025 and 0.014 V, which suggests a partial deviation from Le Chatelier Principle.

Supplementary Note 3:

Additional four repetitive tests (Supplementary Figure 18-19) were performed for the operando Raman under the same condition of Figures 4a, 4b. The weak Raman signal is due to the harsh operando test conditions.[21, 22] In this work, the operando Raman only probes the signals of adsorbed species during catalytic processes. The scattered Raman signals are mainly focused on bond vibrations of adsorbed species (like $*O_2$ and *OOH in ORR) on catalyst surfaces, which are known to be very weak as comparison to bulk materials. To confirm the accuracy of the experimental results, Raman spectrometer software is used to directly determine the peak positions and intensities. On this basis, the Raman information of ER-ZnO are compared at different applied potentials. In general, the intensity of *OOH peaks continued to increase while the intensity of $*O_2$ peaks decrease, which is comparable to the literature,[23-25] and proves the results of our operando Raman test valid for use.

Supplementary Note 4:

N_2 activation has been rarely studied in the literature, which is mainly because of current two-electron ORR studies focusing on high-purity O_2 environment (> 99.9%). In this work, the two-electron ORR performances was investigated in mixed N_2/O_2 environments. The findings would advance the large-scale electrochemical synthesis of hydrogen peroxide in the near future.

Generally, O_2 and N_2 activation at the cathode mainly follows the following equations:[26]

$$2e\text{-}ORR: O_2 + 2H^+ + 2e^- \rightarrow H_2O_2 \left(E^o = 0.68\ V\ vs\ RHE,\ in\ neutral\right)$$

$$NRR: N_2 + 6H_2O + 6e \rightarrow 2NH_3 + 6OH^- \left(E^0 = 0.227\ V\ vs.\ RHE,\ in\ neutral\right)$$

Obviously, the theoretical potentials of 2e-ORR and NRR only shows limited gap (0.453 V), so in most cases it is necessary to consider both reactions.

Experimentally, flow-cell systems were utilized to achieve industrial-grade current densities (Figure 3b). In air environment (21% O_2), the applied potentials on the ER-ZnO catalyst at 50 ~ 300 mA cm^{-2} current densities are 0.21 ~ -0.67 V. On the other hand, at the current density of 200 mA cm^{-2}, the applied potentials on the ER-ZnO catalyst are -0.27 V (100% O_2), -0.24 V (80% O_2), -0.25 V (40% O_2) and -0.36 V (21% O_2), respectively. Therefore, both 2e-ORR and NRR may occur simultaneously under such potential range.

Accordingly, the effect of N_2 activation is investigated by using TPD (Supplementary Figure 21), operando Raman spectra (Figure 4a-c) and DFT calculations (Figure 4d-g). In the TPD experiments, the adsorption capacity of ER-ZnO (0.104) on N_2 was less than that of ZnO (0.167, Supplementary Figure 21). Similarly, the vibrational peaks of $*N_2$ were tested on operando Raman, showing much smaller peak intensity of ER-ZnO as comparison to ZnO (Supplementary Figure 20). The adsorption of N_2 on the catalyst surface was calculated by using DFT, showing the smaller adsorption energy of ER-ZnO as comparison to ZnO (-0.34 vs -1.25 eV, Figure 4d).

Supplementary Note 5:

The E-R mechanism has not been extensively studied in two-electron ORR. This is primarily because of present studies seldom exploring the impact of proton sources on 2e-ORR activities. In this work, we have carefully examined the intermediate processes of ZnO and ER-ZnO for 2e-ORR. For the first time, we have found the proton sources that have a significant influence on the 2e-ORR activities, particularly under industrial-level current densities. Our findings may promote the industrialization of electrochemical hydrogen peroxide production.

More specifically, based on the literature and our experimental results, the reaction intermediate process of 2e-ORR is as follows: O_2 + * → *O_2 → *OOH → *H_2O_2 → * + H_2O_2. According to the literature,[27] the first protonation process is considered as the decisive step (*O_2 → *OOH), which is the focus of our study.

We have then utilized heterogeneous catalysis to investigate the reaction mechanism of the hydrogenation process (*O_2 → *OOH). The heterogeneous catalysis theory typically involves three mechanisms: Langmuir-Hinshelwood (L-H) mechanism, Eley-Rideal (E-R) mechanism, and Mars-van Krevelen (M-V) mechanism. Because of M-V mechanism mainly occurring on anion-deficient catalysts,[28] we then only examine the L-H mechanism and E-R mechanism. According to the literature,[29] classical L-H mechanism can be described as (Supplementary Figure 36): both reactant gas molecules (A and B) adsorbed on the surface of catalyst (*A and *B), followed by the coupling of adjacent *A and *B to form the final product of *AB. On the other hand, the E-R mechanism requires the adsorption of a single reactant gas molecule (*e.g.*, A), followed by *A on the surface combining with the free gaseous molecule B in the surrounding environment to form the final product of *AB.

We have noted the traditional E-R mechanism mainly occurring in the solid-gas phase and is dependent on the catalyst structure and properties.[30] In this paper, we have updated E-R mechanism according to the source of reactant: the gaseous A is firstly adsorbed on the catalyst surface to form *A, followed by the free species B in

the liquid phase directly combining with *A at triple-phase interface, finally forming the product of *AB.

We have further confirmed the updated E-R mechanism through a series of experiments (such as *operando* Raman and temperature programmed desorption, Figure 4a-c) and theoretical calculations (Figure 4d-g). Particularly in theoretical calculations, we have plotted the kinetic barrier of E-R and L-H mechanisms on ER-ZnO and ZnO. As shown in Figure 4g and Supplementary Figure 39, ER-ZnO prefers E-R than L-H mechanisms because of smaller energy barriers (-0.67 vs 0.74 eV), which is different from ZnO (1.29 vs 0.38 eV).

Supplementary Note 6:

Firstly, the structural optimization by theoretical simulations (Supplementary Figures 24-25) shows that $*O_2$ on ER-ZnO is a linear adsorption style due to the presence of defects, while the bridging adsorption style on ZnO surface. The previous literature suggests that $*O_2$ linear adsorption favors the two-electron ORR route, whereas bridge adsorption favors the four-electron route.[31]

Secondly, according to Sabatier's principle, reaction selectivity is sensitive to the appropriate adsorption energy. The adsorption of the key *OOH intermediate can have a negative impact on ORR activities if it is either too strong or too weak. If the adsorption of *OOH is too strong, the O-O bond will break, leading to a reaction that favors the four-electron pathway. Conversely, if the *OOH adsorption strength is too weak, the intermediate cannot be stabilized, resulting in low reaction activity. According to the calculation data presented in Figure 4d, the adsorption energy of *OOH on ER-ZnO is appropriate (-1.51 eV), indicating intermediate stability on the surface of ER-ZnO. Nevertheless, the adsorption energy of *OOH on ER-ZnO is not as strong as that on ZnO (-2.62 eV). Therefore, ER-ZnO prefers two-electron ORR while ZnO prefers four-electron ORR, which is consistent with the experimental data (Figure 1, 3).

Additional calculations of four-electron ORR diagrams on ER-ZnO have been conducted (Supplementary Figures 40-41). The data indicates that four-electron ORR is less likely to occur on the ER-ZnO surface compared to ZnO due to a large positive free energy change after the production of *OOH (0.28 eV vs. -1.47 eV).

Supplementary Note 7:

The O_2 partial pressure indeed has significant effects on the kinetics of two-electron ORR. In electrochemical reactions, the B-V and Levich equations are generally used to describe reaction kinetics:[32]

$$j_k = j_0\left[-\exp\left(\frac{-\alpha nF}{RT}\eta\right)+\exp\left(\frac{(1-\alpha)nF}{RT}\eta\right)\right]$$

$$i_1 = 0.62nAFD^{2/3}\omega^{1/2}\mu^{-1/6}C_0^*$$

Accordingly, the current density and O_2 concentration (C_0) have the following first order kinetics relationship:

$$\frac{1}{J}=\frac{1}{J_k}+\frac{1}{0.62nAFD^{2/3}\omega^{1/2}\mu^{-1/6}C_0^*}$$

Furthermore, the surface coverage of reaction intermediates can be affected by the partial pressure of O_2. As demonstrated in Figures 4a and 4b in the main text, operando Raman spectroscopy was used to examine the adsorption of *OOH intermediates on ZnO and ER-ZnO under 100% ~ 21% O_2 environments. The coverage of *OOH on the surface of ZnO change significantly, while on the ER-ZnO remains consistent. It is worth noting that the coverage of *OOH on the surface of ER-ZnO was significantly greater than that of ZnO in 21% O_2. The high coverage of intermediates, as reported in the literature[33], favors a low energy barrier for the $*O_2 \rightarrow$ *OOH step, resulting in a fast reaction rate and consequently high reaction selectivity.

To further investigate the effect of O_2 partial pressure on activities, the two-electron ORR performance of ER-ZnO were tested at lower O_2 partial pressures (21% ~ 5%, Supplementary Figure 43-45). The limiting diffusion current densities are calculated for different concentration mixtures across the electrode surface according to the following equation[34]:

$$\mathrm{I_d} = \frac{nFD_{ON}\prod}{\delta}In\frac{\prod}{c_N}$$

The limiting diffusion current density (I_d) (mA cm^{-2}) is determined by the gas reactant flow (∏) (mol cm^{-3}), the diffusion coefficient of oxygen in natural air (D_{ON})

(cm^2 s^{-1}), and the concentration of nitrogen on the surface of the gaseous diffusion layer (c_N) (mol cm^{-3}). Additionally, the electron reaction numbers (n), the Faraday constant (F) (96485 C mol^{-1}), and the thickness of the gaseous diffusion layer (δ) (cm) are also considered.

Obviously, the limiting diffusion current densities of the reaction decrease continuously with O_2 partial pressures. Notably, The ER-ZnO catalyst can maintain the current density close to the theoretical limits under different partial pressures (Supplementary Figure 46).

3. Supplementary Figures

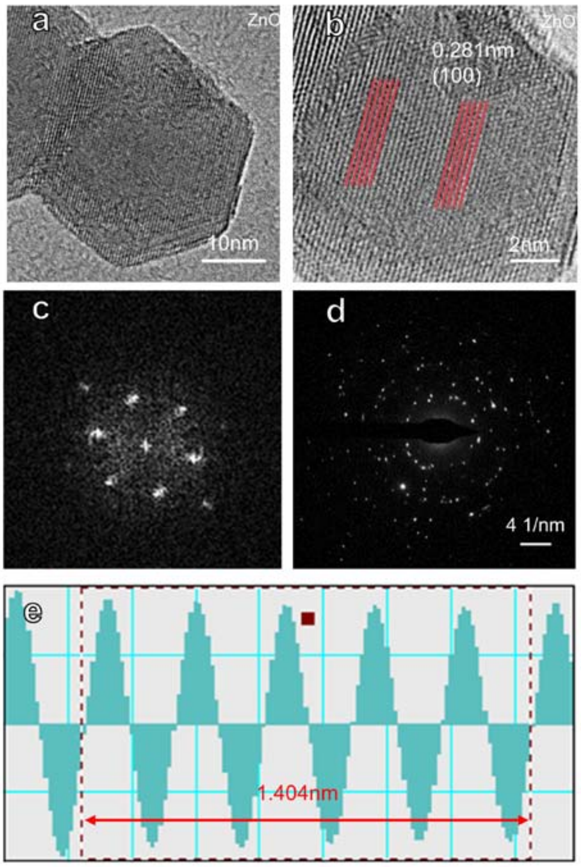

Supplementary Figure 1. **Structural characterizations of the reference ZnO. a**, **b,** HR-TEM image. **c**, FFT image of HR-TEM. **d**, SAED image of HR-TEM. **e**, The intensity profile of (100) lattice plane.

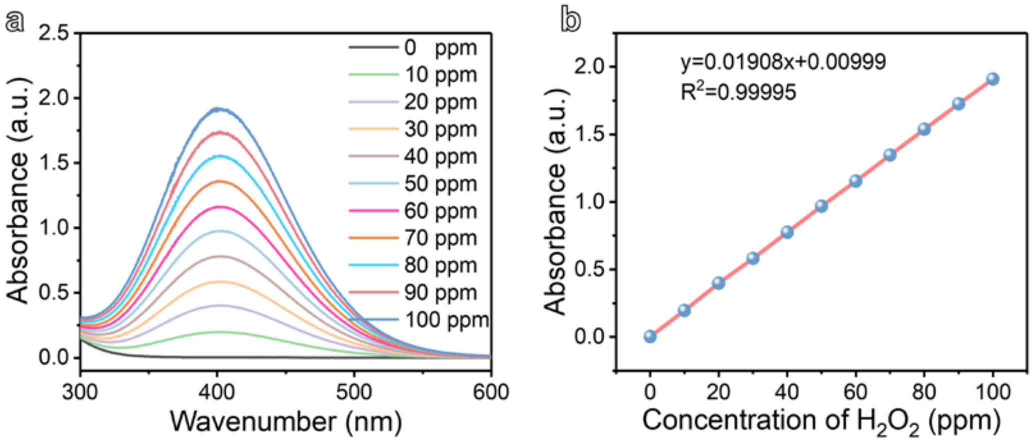

Supplementary Figure 2. **Standard curves for quantifying H_2O_2**. **a**, UV-vis spectra of Ti^{4+} solution with various concentrations. **b**, The corresponding standard curve.

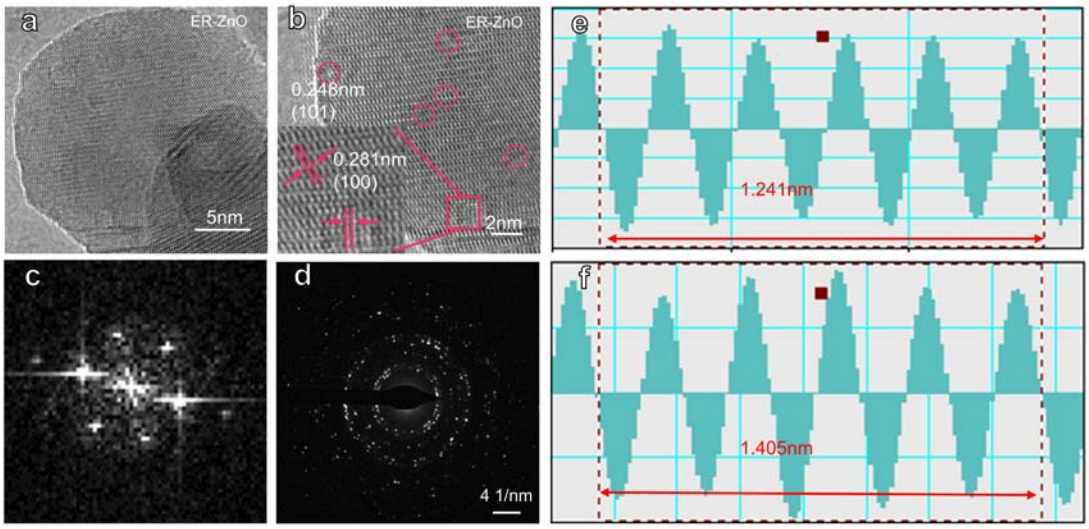

Supplementary Figure 3. Structural characterizations of ER-ZnO. a, **b,** HR-TEM image. **c**, FFT image of HR-TEM. **d**, SAED image of HR-TEM. **e**, **f,** The intensity profiles of (100) and (101) lattice planes.

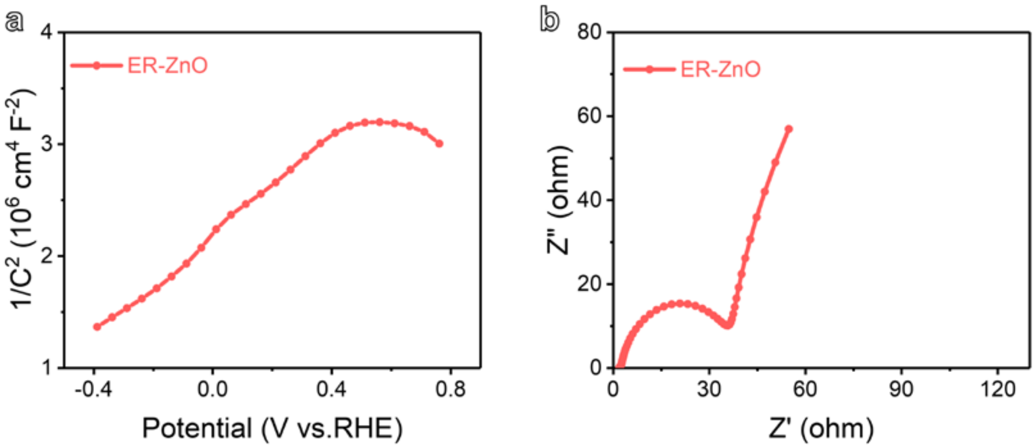

Supplementary Figure 4. **Electrochemical properties of ER-ZnO.** **a**, Mott-Schottky curves. **b**, electrochemical impedance spectroscopy (EIS) measurement.

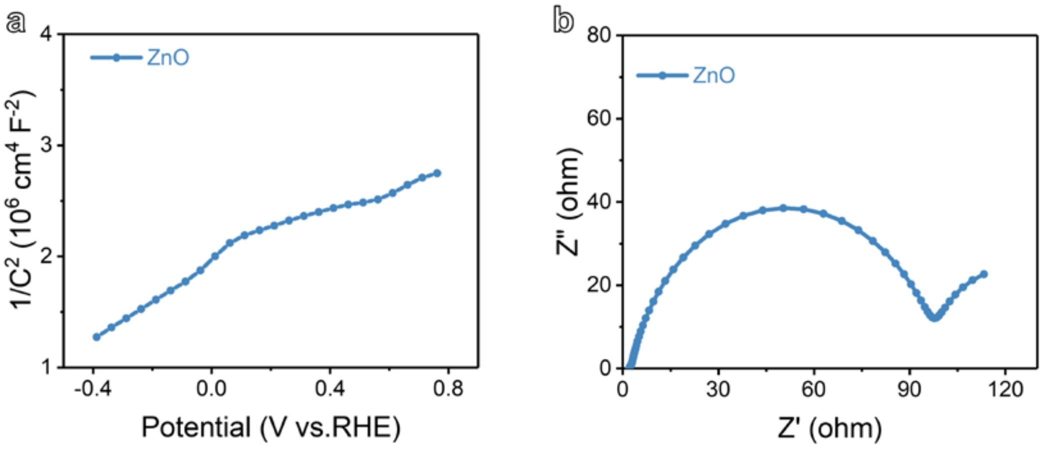

Supplementary Figure 5. **Electrochemical properties of ZnO. a**, Mott-Schottky curves. **b**, electrochemical impedance spectroscopy (EIS) measurement.

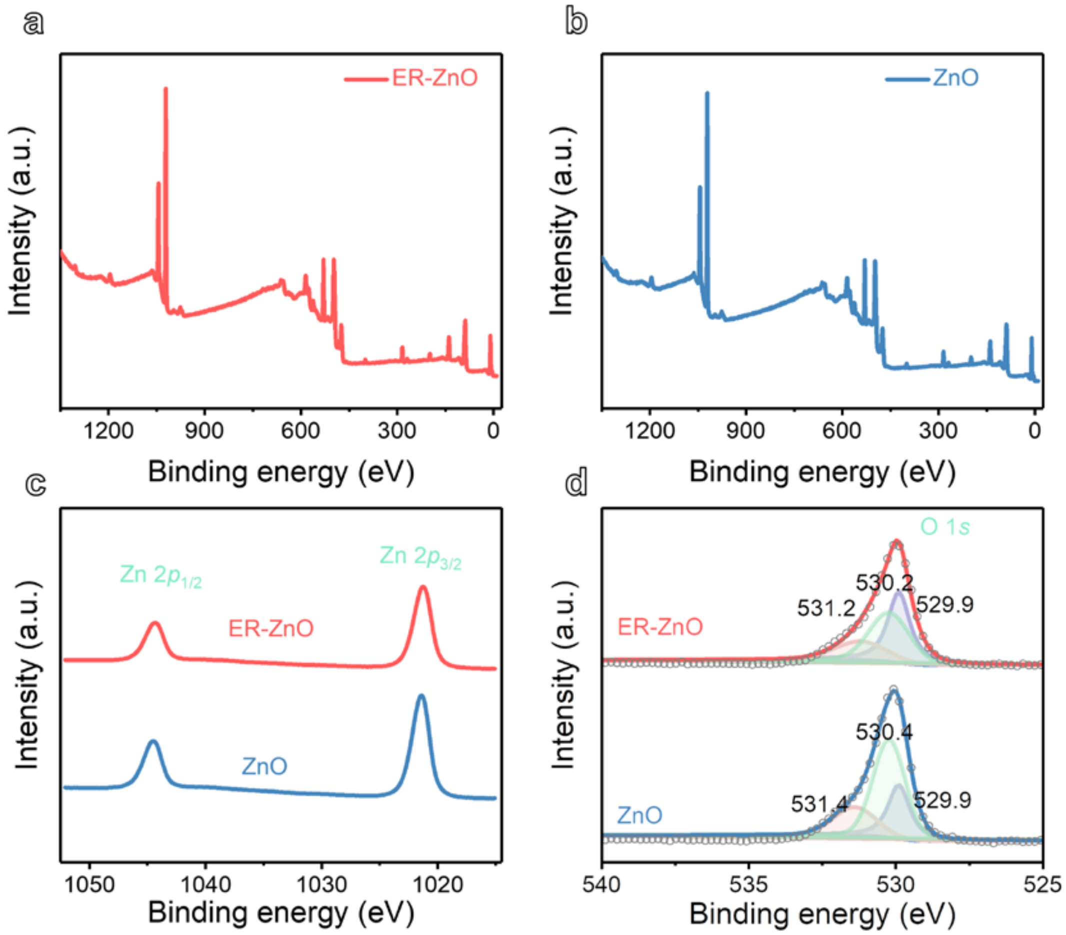

Supplementary Figure 6. XPS characterization analysis of ER-ZnO and reference ZnO. a, XPS survey of ER-ZnO. **b**, XPS survey of ZnO. **c**, Zn 2p characteristic peaks of ZnO and ER-ZnO. **d**, O 1s characteristic peaks of ZnO and ER-ZnO.

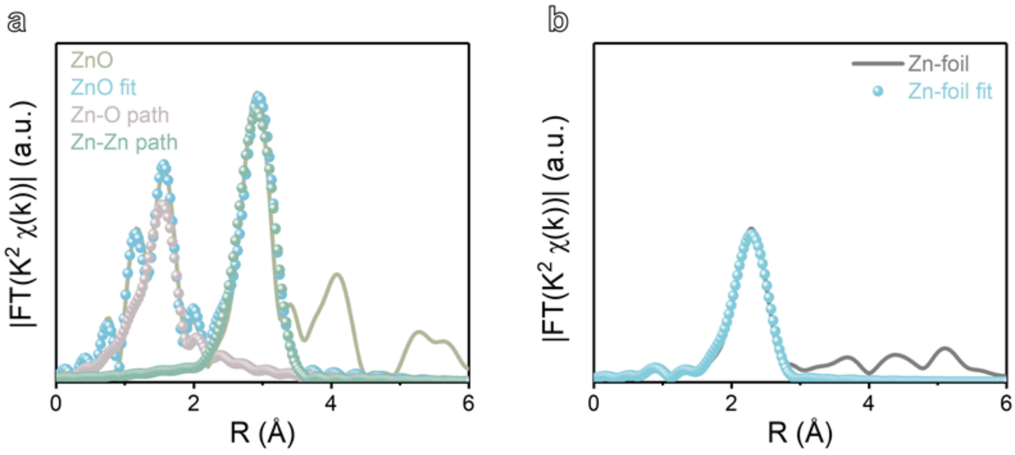

Supplementary Figure 7. **EXAFS curve and the fitting lines of reference ZnO and Zn-foil.** **a**, EXAFS curve and the fitting lines of ZnO. **b**, EXAFS curve and the fitting lines of Zn-foil.

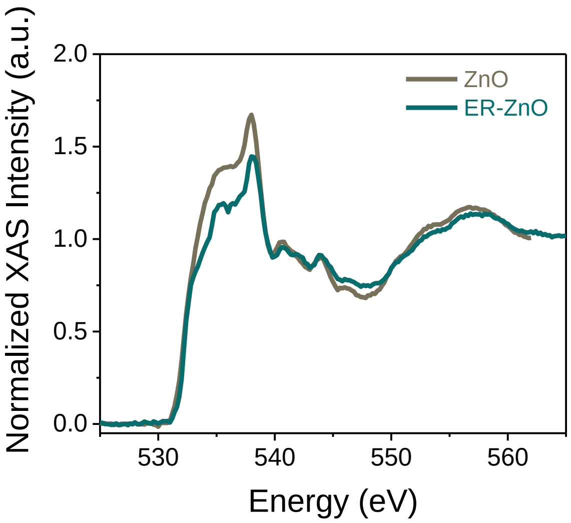

Supplementary Figure 8. **The O k-edge XANES of ER-ZnO and ZnO.** The O element in ER-ZnO and ZnO was detected by soft XAS to demonstrate the electron states.

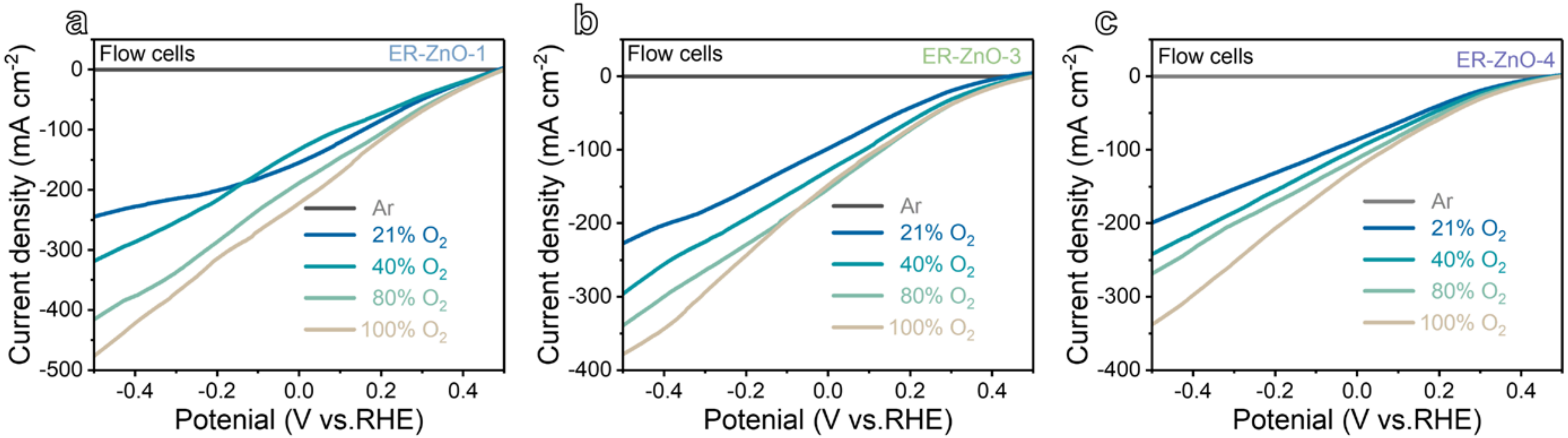

Supplementary Figure 9. **The linear sweep voltametry curves in Ar and mixed O_2 media for a**, reference ER-ZnO-1 catalysts. **b**, reference ER-ZnO-3 catalysts. **c**, reference ER-ZnO-4 catalysts. (prepared from the same procedure to ER-ZnO excepting of adding 1, 3 and 4 mL of glycerol modifier for ER-ZnO-1, ER-ZnO-3 and ER-ZnO-4, respectively).

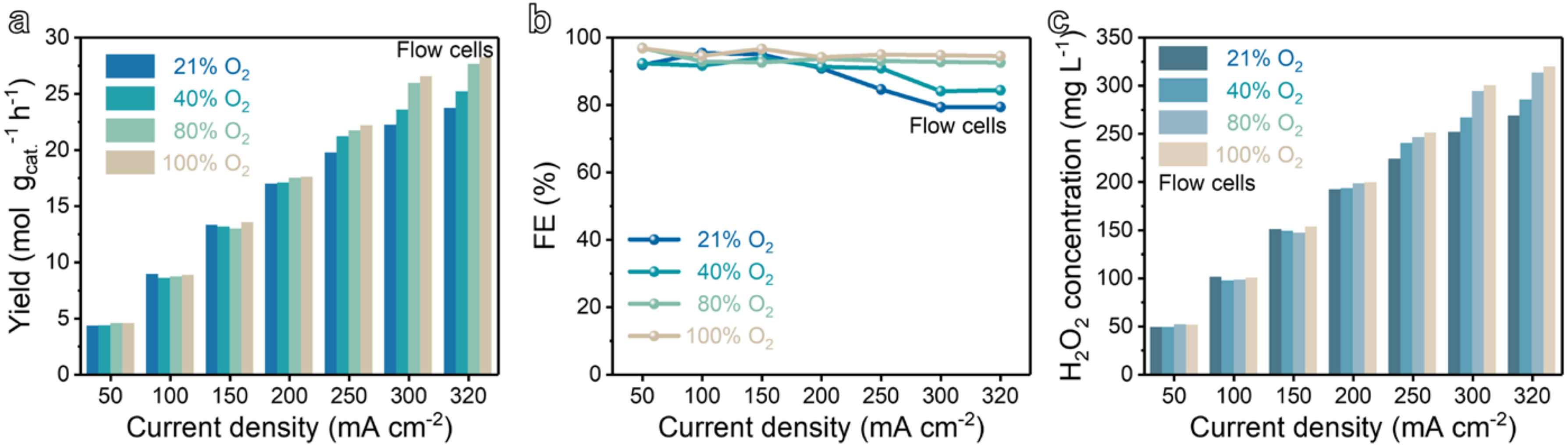

Supplementary Figure 10. **The ORR performances of ER-ZnO-1** (prepared from the same procedure to ER-ZnO excepting of adding 1 mL of glycerol modifier) in mixed O_2 media. **a**, Yield rates. **b,** The corresponding Faradaic efficiencies. **c,** The corresponding H_2O_2 concentration.

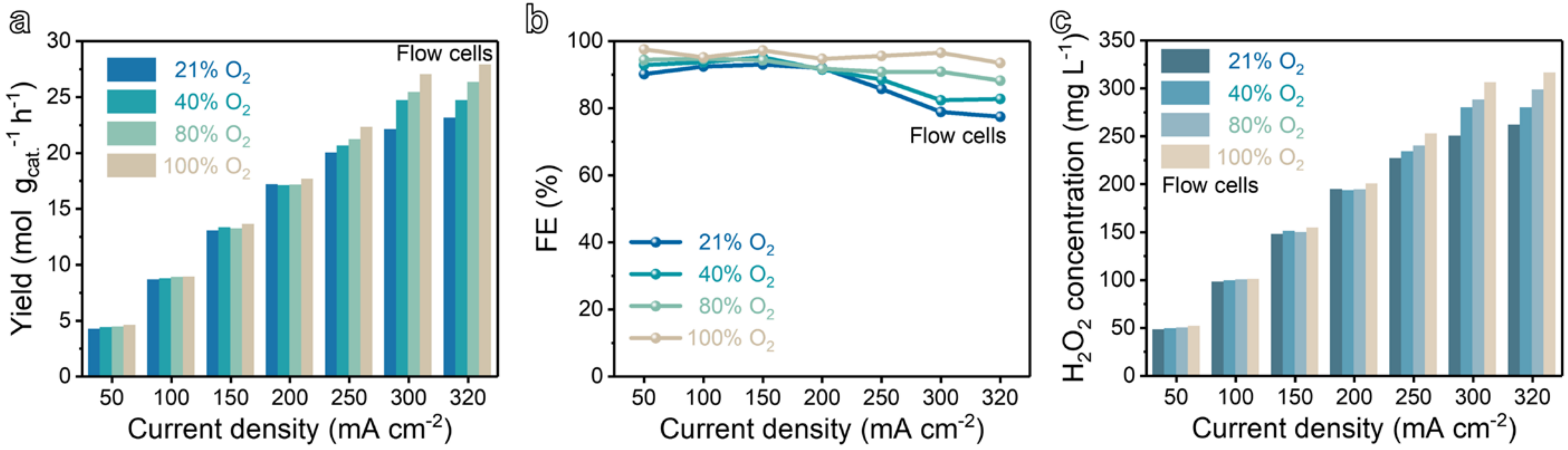

Supplementary Figure 11. **The ORR performances of ER-ZnO-3** (prepared from the same procedure to ER-ZnO excepting of adding 3 mL of glycerol modifier) in mixed O_2 media. . **a**, Yield rates. **b,** The corresponding Faradaic efficiencies. **c,** The corresponding H_2O_2 concentration.

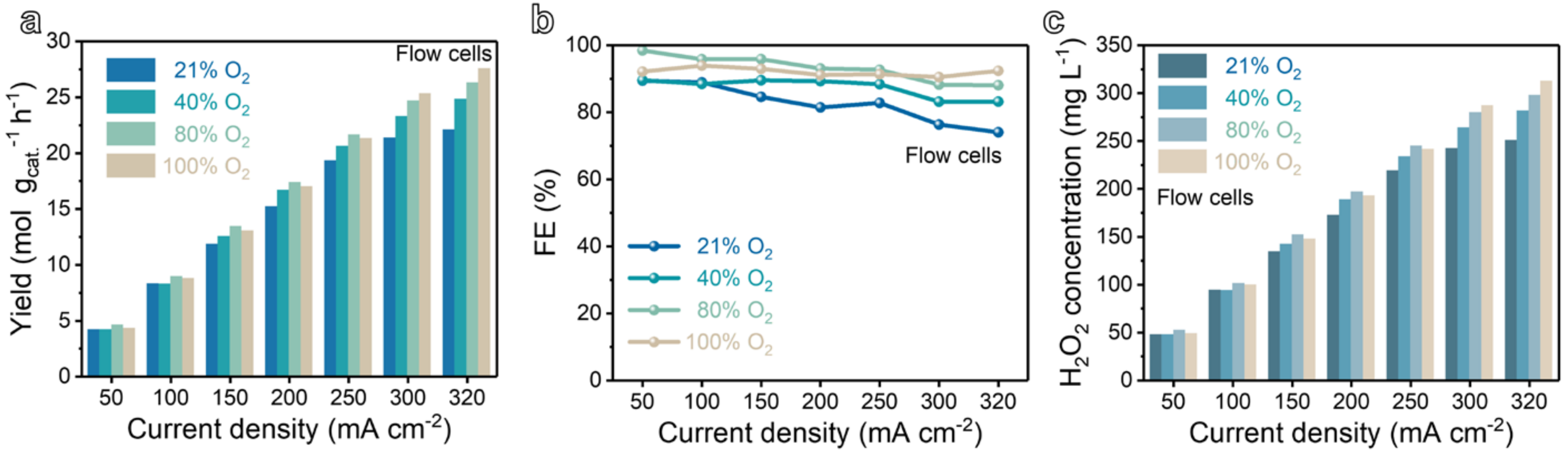

Supplementary Figure 12. **The ORR performances of ER-ZnO-4** (prepared from the same procedure to ER-ZnO excepting of adding 4 mL of glycerol modifier) in mixed O_2 media. . **a**, Yield rates. **b,** The corresponding Faradaic efficiencies. **c,** The corresponding H_2O_2 concentration.

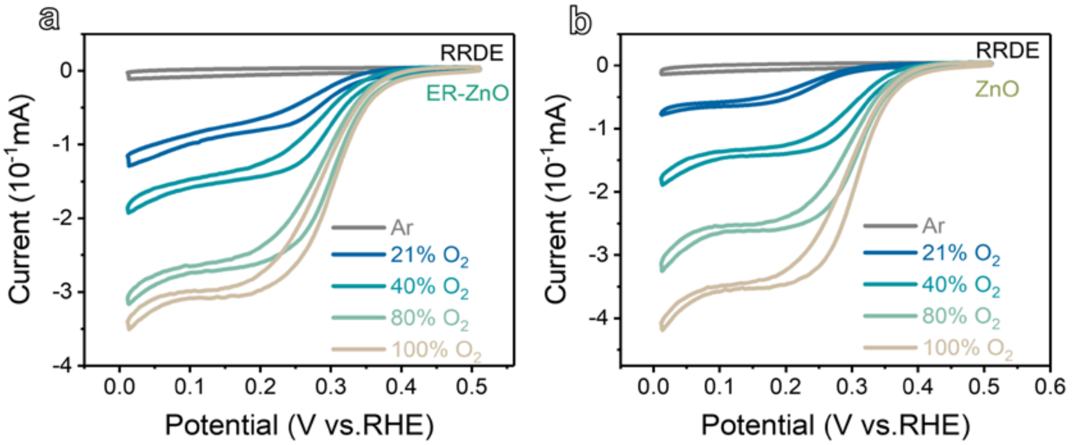

Supplementary Figure 13. **The CV curves in Ar and mixed O_2 media in RRDE system for a**, ER-ZnO. **b,** ZnO.

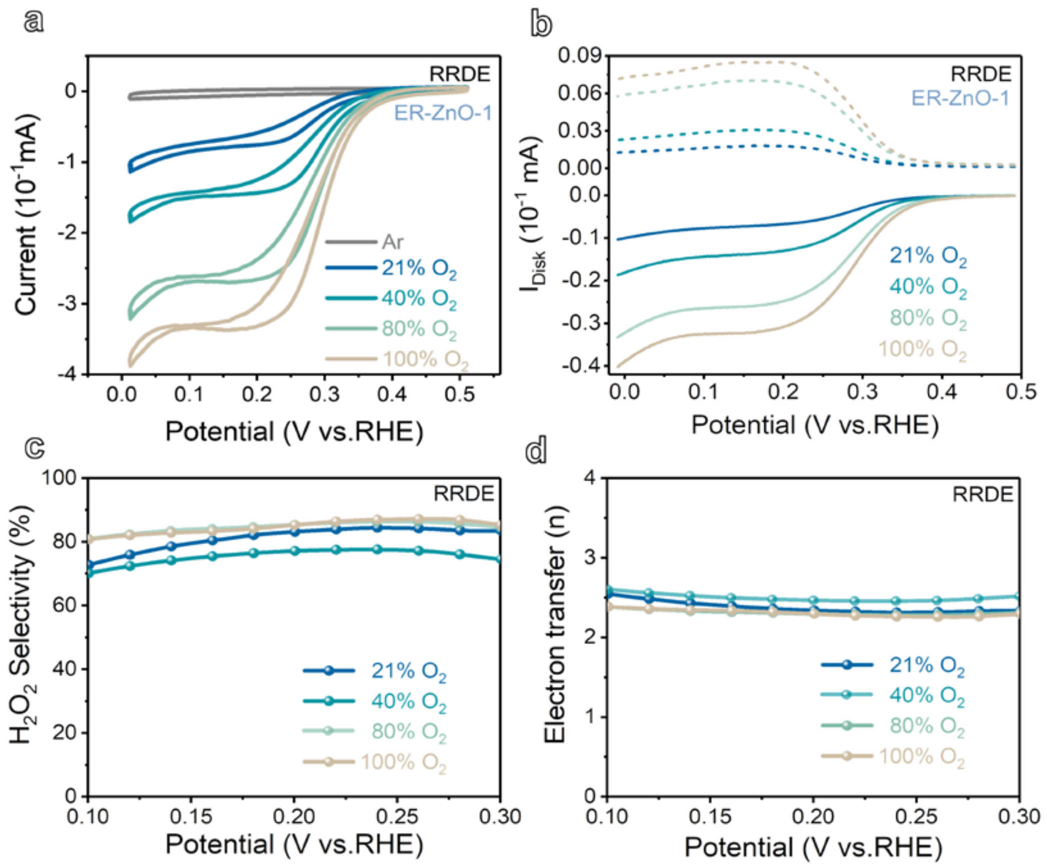

Supplementary Figure 14. **Electrochemical characterizations of ER-ZnO-1** (prepared from the same procedure to ER-ZnO excepting of adding 1 mL of glycerol modifier) in RRDE system. **a**, The CV curves in Ar and mixed O_2 media. **b,** The LSV polarization curve, and the detected H_2O_2 current with a ring constant potential of 1.2 V. **c**, **d**, the corresponding H_2O_2 molar fraction selectivity and electron transfer number (n).

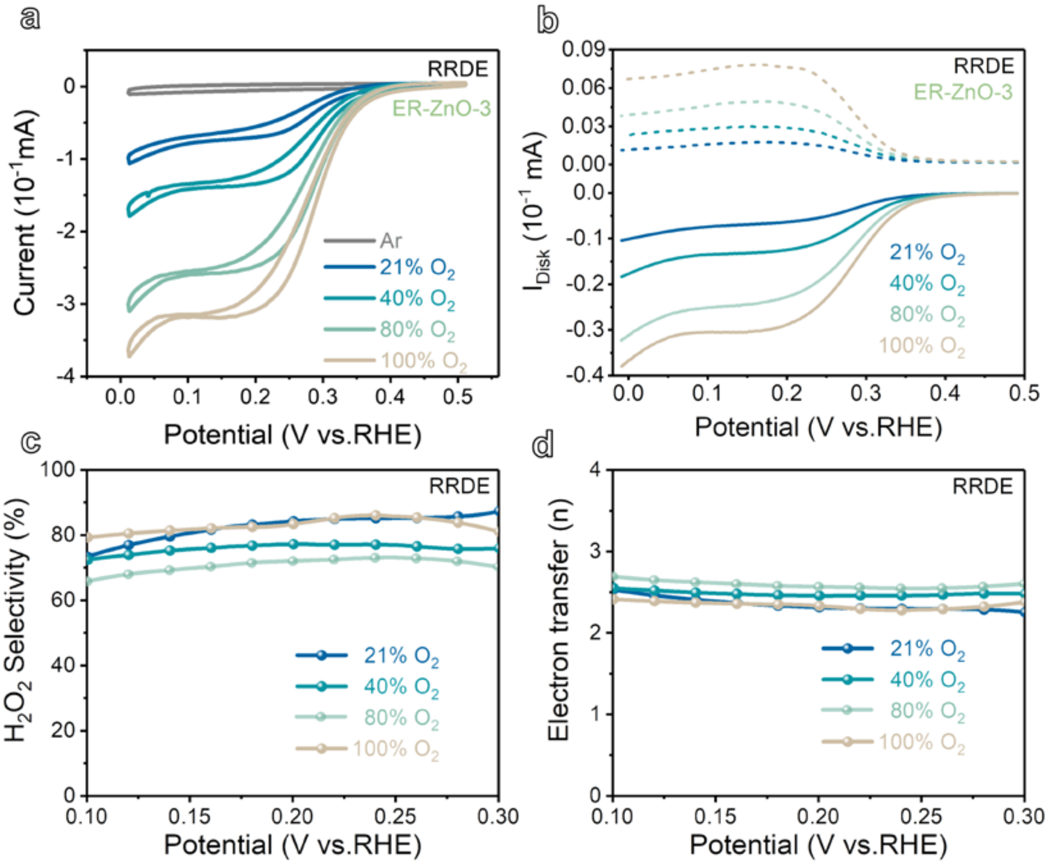

Supplementary Figure 15. **Electrochemical characterizations of ER-ZnO-3** (prepared from the same procedure to ER-ZnO excepting of adding 3 mL of glycerol modifier) in RRDE system. **a**, The CV curves in Ar and mixed O_2 media. **b,** The LSV polarization curve, and the detected H_2O_2 current with a ring constant potential of 1.2 V. **c**, **d**, the corresponding H_2O_2 molar fraction selectivity and electron transfer number (n).

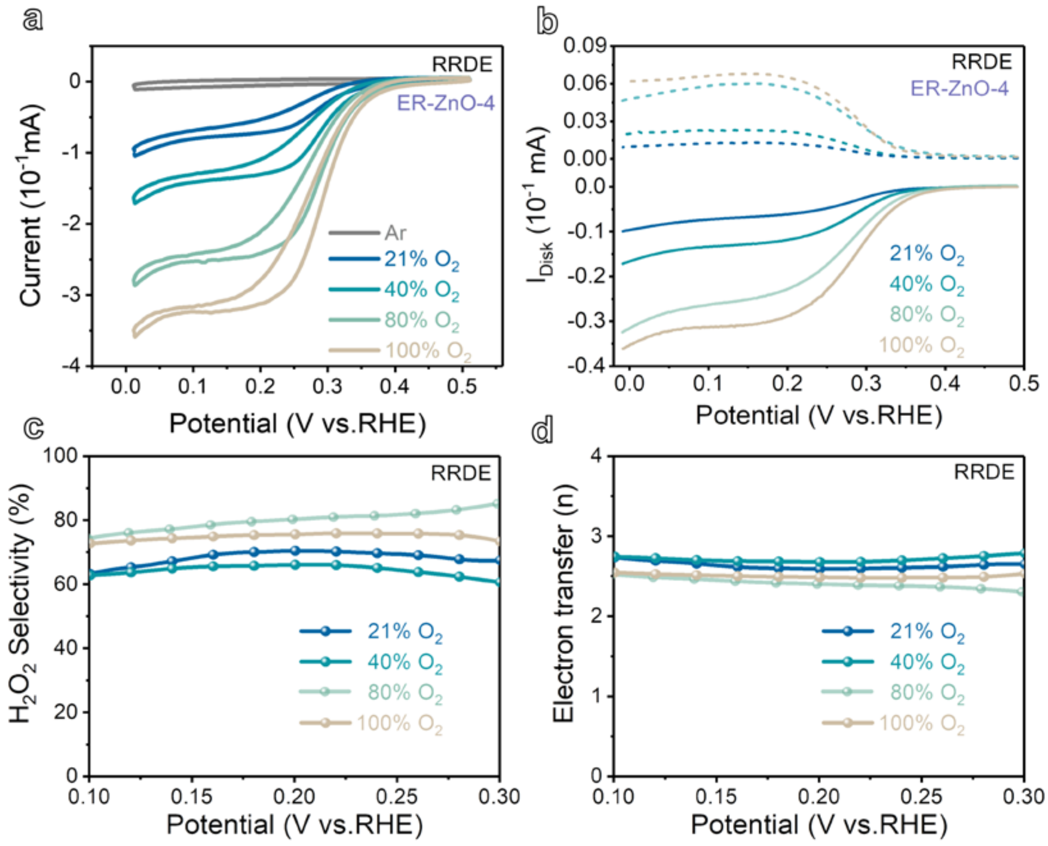

Supplementary Figure 16. **Electrochemical characterizations of ER-ZnO-4** (prepared from the same procedure to ER-ZnO excepting of adding 4 mL of glycerol modifier) in RRDE system. **a**, The CV curves in Ar and mixed O_2 media. **b,** The LSV polarization curve, and the detected H_2O_2 current with a ring constant potential of 1.2 V. **c**, **d**, the corresponding H_2O_2 molar fraction selectivity and electron transfer number (n).

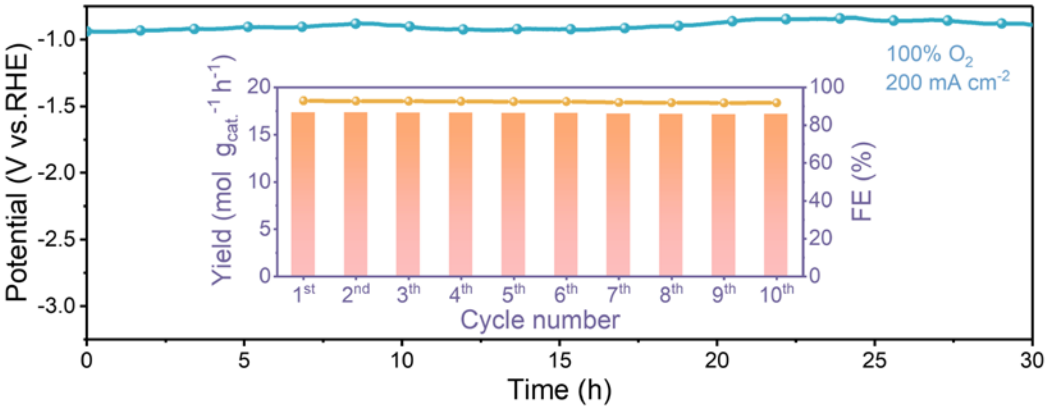

Supplementary Figure 17. **The durability test of ER-ZnO** in 100% O_2 atmosphere at 200 mA cm^{-2} for 30 hrs. Inset is the yield and corresponding Faradaic efficiencies among 10-times chronoamperometry cycles at 200 mA cm^{-2}.

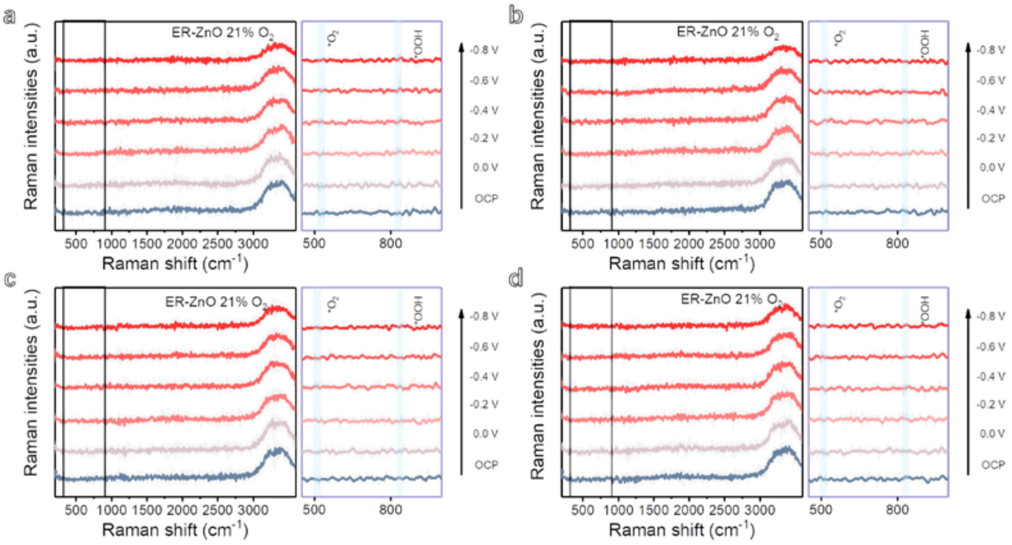

Supplementary Figure 18. **Four repetitive operando Raman tests for ER-ZnO in 21% O_2 under the same condition**, **a**, first test. **b,** section test. **c**, third test. **d**, fourth test. Additional four repetitive tests were performed for the operando Raman under the same condition to confirm the accuracy of the experimental results.

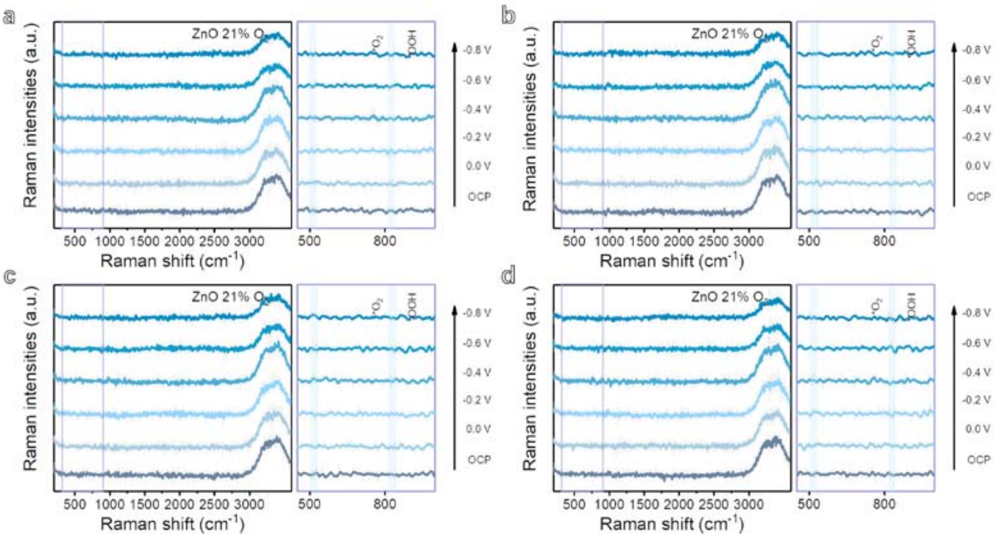

Supplementary Figure 19. **Four repetitive operando Raman tests for ZnO in 21% O_2 under the same condition**, **a**, first test. **b,** section test. **c**, third test. **d**, fourth test. Additional four repetitive tests were performed for the operando Raman under the same condition to confirm the accuracy of the experimental results.

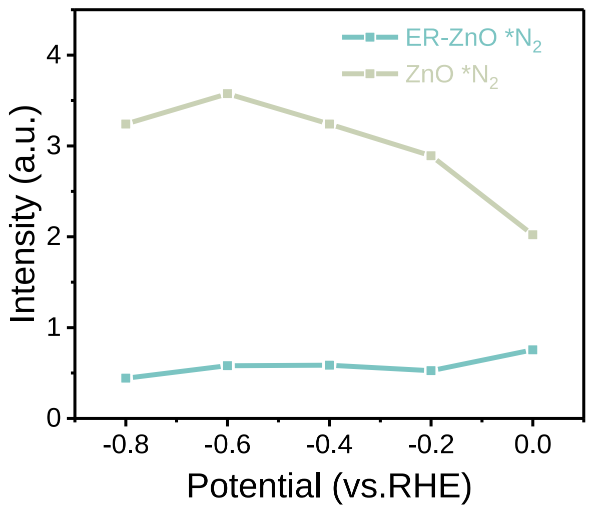

Supplementary Figure 20. **The peak intensity of *N_2 in operando Raman test.** The Raman peak at 2325 cm^{-1} is attributed to N-N stretching-vibrational mode of N_2 molecules. The peak intensities for ER-ZnO and ZnO in different potentials were collected to illustrate the effect of adsorped *N_2.

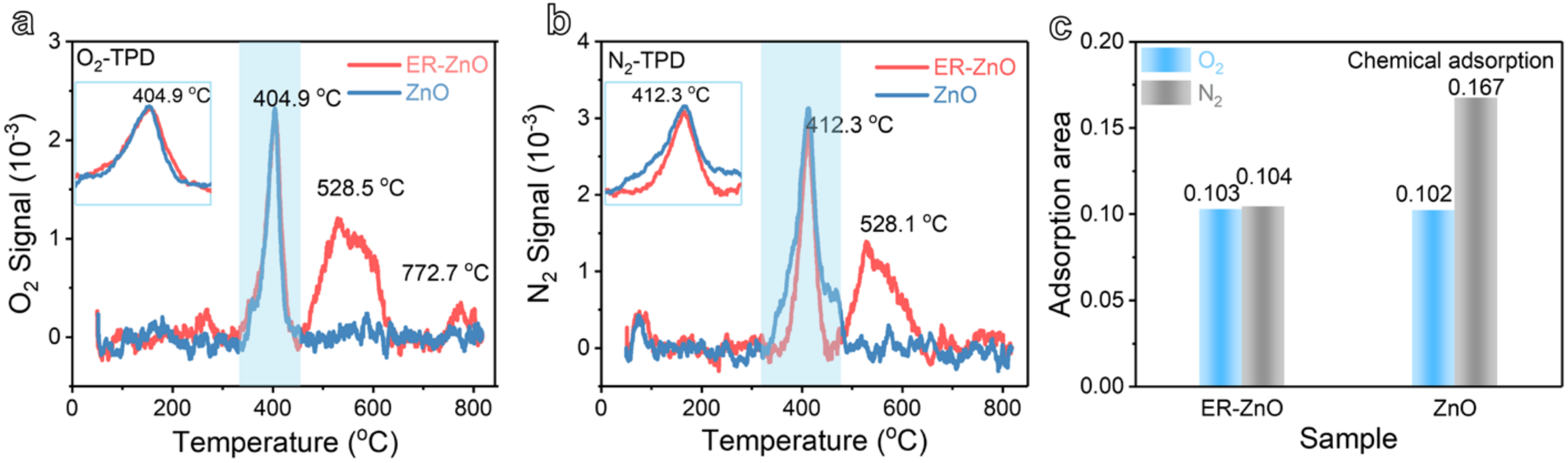

Supplementary Figure 21 The O_2-TPD and N_2-TPD data of reference ZnO and ER-ZnO. a, The O_2-TPD data of ZnO and ER-ZnO, where the signals at 404.9 and 528.5 ^{o}C are attributable to surface adsorbed O_2 and lattice O_2, respectively. **b**, The N_2-TPD data of ZnO and ER-ZnO, where the signals at 412.3 and 528.1 ^{o}C are attributable to surface adsorbed N_2 and lattice N_2, respectively. **c**, the comparison of surface adsorption areas for ZnO and ER-ZnO in O_2 and N_2-TPD profiles.

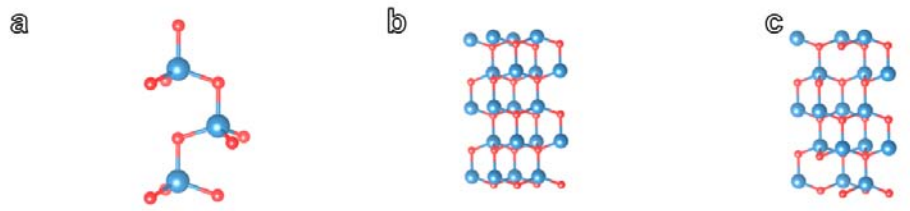

Supplementary Figure 22 The unit cell structure of reference ZnO and ER-ZnO.

a, The unit cell structure of ZnO. **b**, the supercell structure of ZnO. **c**, the supercell structure of ER-ZnO with zinc vacancy.

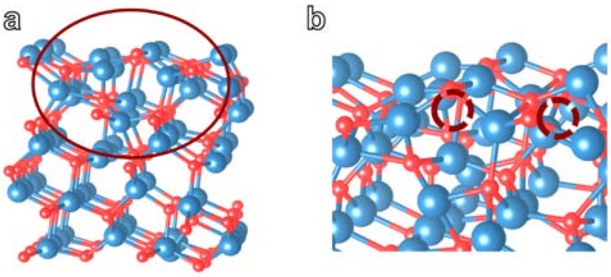

Supplementary Figure 23. The local structures of ER-ZnO. a, **b,** The local structures of ER-ZnO, where the zinc defects are clearly marked.

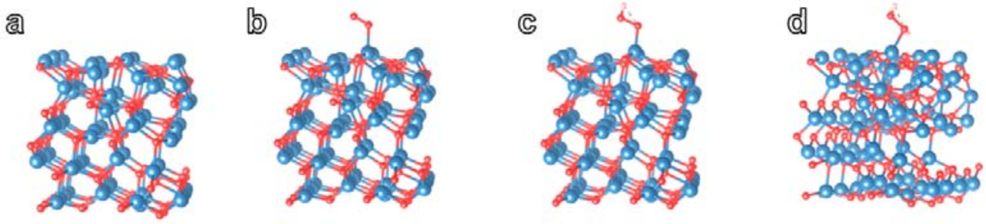

Supplementary Figure 24. The optimized supercell structure of ER-ZnO absorbed by different ORR intermediates (None for **a**; *O_2 for **b**; *OOH for **c**; *HOOH for **d**).

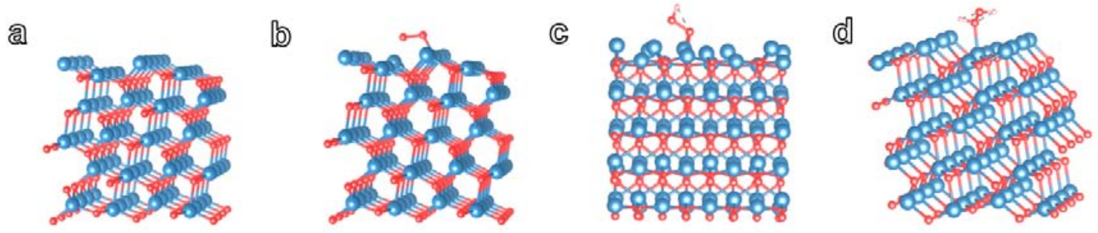

Supplementary Figure 25. The optimized supercell structure of the reference ZnO absorbed by different ORR intermediates (None for **a**; *O_2 for **b**; *OOH for **c**; *HOOH for **d**).

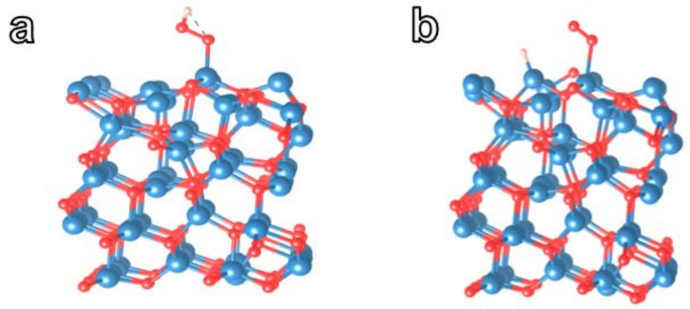

Supplementary Figure 26. The structure of intermediate for ER-ZnO following E-R and L-H pathway. a, the structure of *OOH intermediate for ER-ZnO following E-R pathway. **b**, the structure of *O_2-*H intermediate for ER-ZnO following L-H pathway.

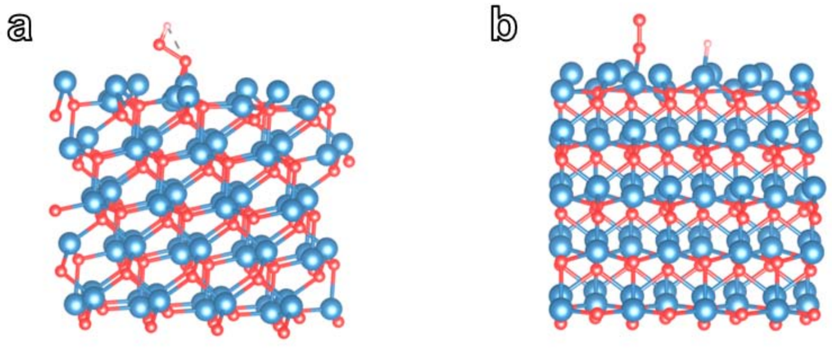

Supplementary Figure 27. The structure of intermediate for ZnO following E-R and L-H pathway. a, the structure of *OOH intermediate for the reference ZnO following E-R pathway. **b**, the structure of *O_2-*H intermediate for the reference ZnO following L-H pathway.

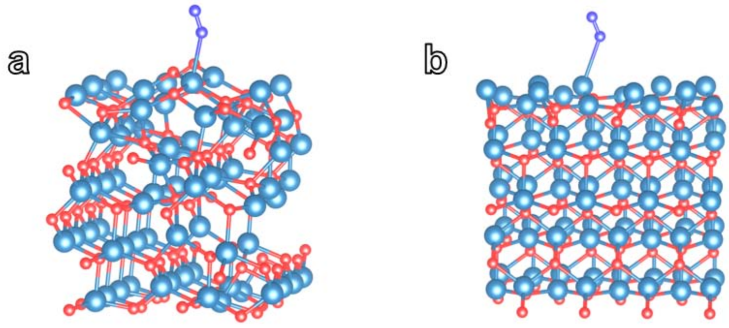

Supplementary Figure 28. The optimized supercell structure of ER-ZnO and ZnO absorbed by N_2 (ER-ZnO *N_2 for **a**; ZnO *N_2 for **b**)

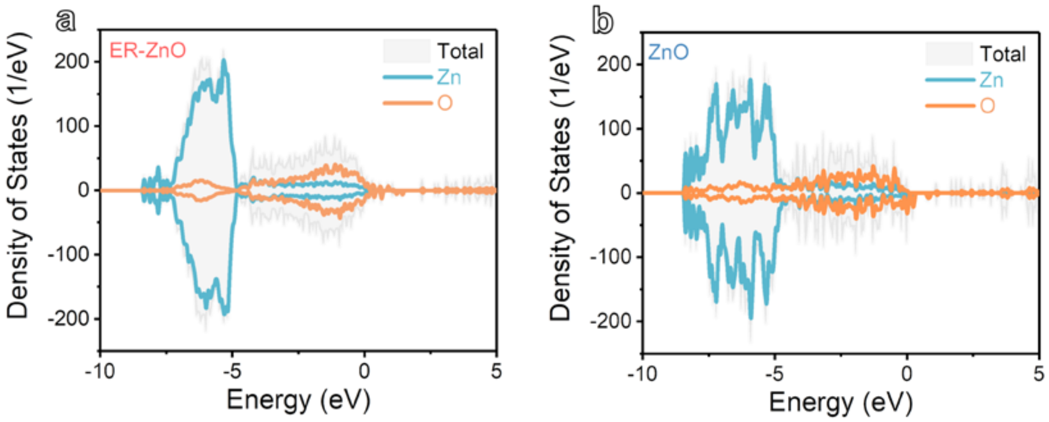

Supplementary Figure 29. Density of states (DOS) of a, ER-ZnO, and **b**, ZnO.

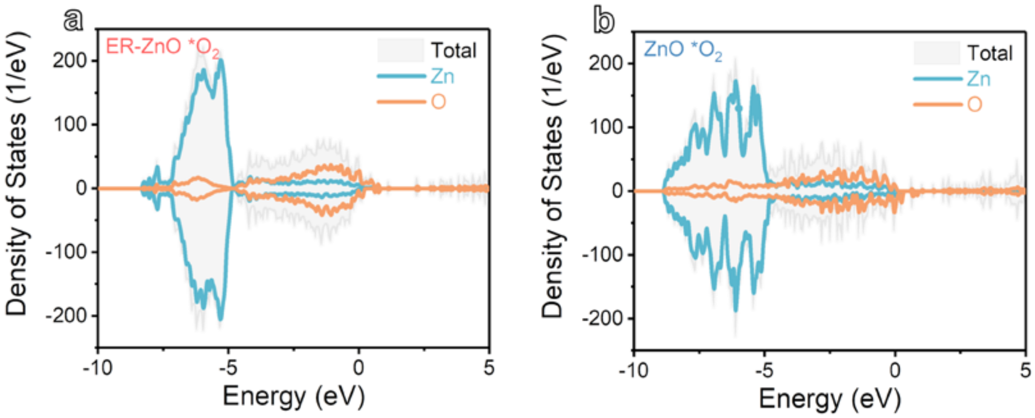

Supplementary Figure 30. Density of states (DOS) of a, ER-ZnO adsorbed by $*O_2$, and the reference ZnO adsorbed by $*O_2$.

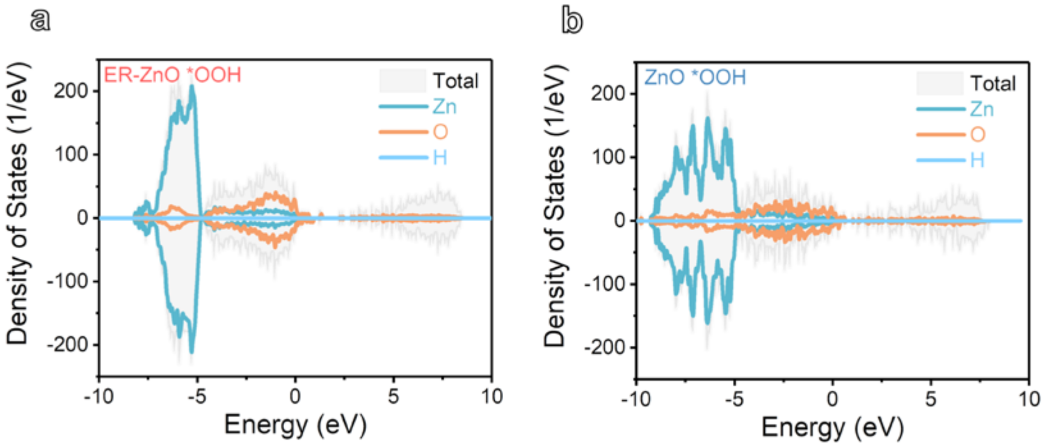

Supplementary Figure 31. Density of states (DOS) of **a**, ER-ZnO adsorbed by *OOH, and **b**, the reference ZnO adsorbed by *OOH.

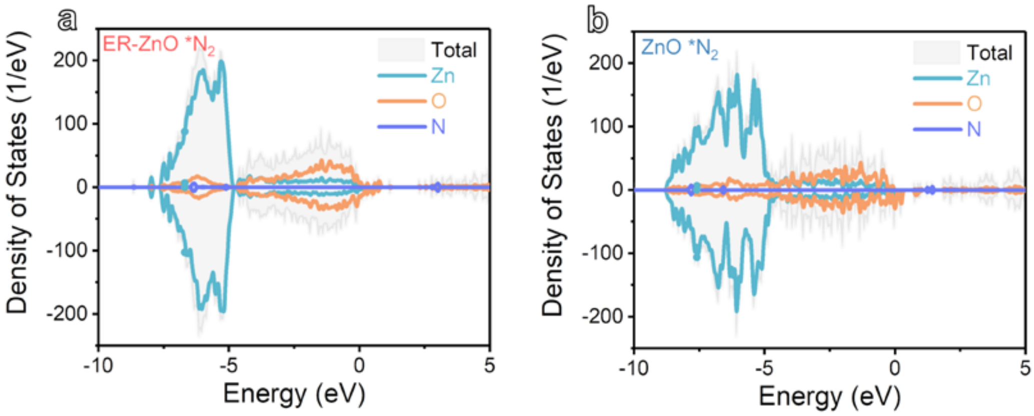

Supplementary Figure 32. Density of states (DOS) of a, ER-ZnO adsorbed by $*N_2$, and **b**, the reference ZnO adsorbed by $*N_2$

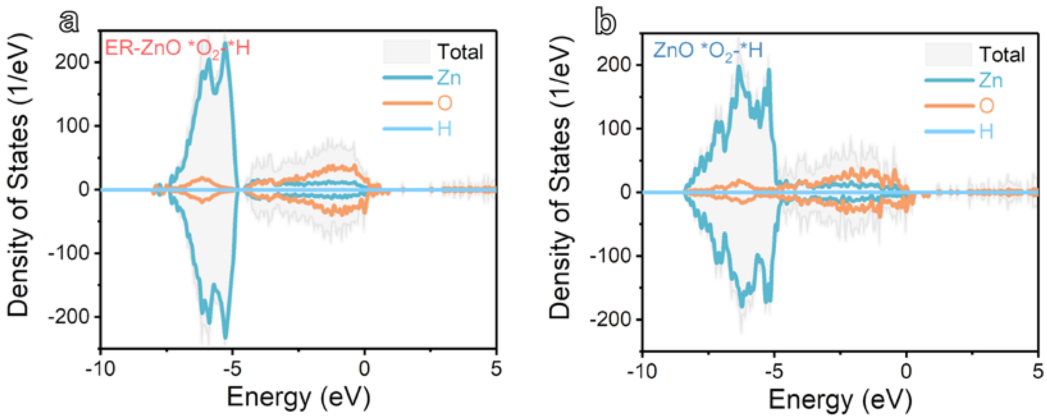

Supplementary Figure 33. Density of states (DOS) of a, ER-ZnO adsorbed by $*O_2$-*H, and **b**, the reference ZnO adsorbed by $*O_2$-*H.

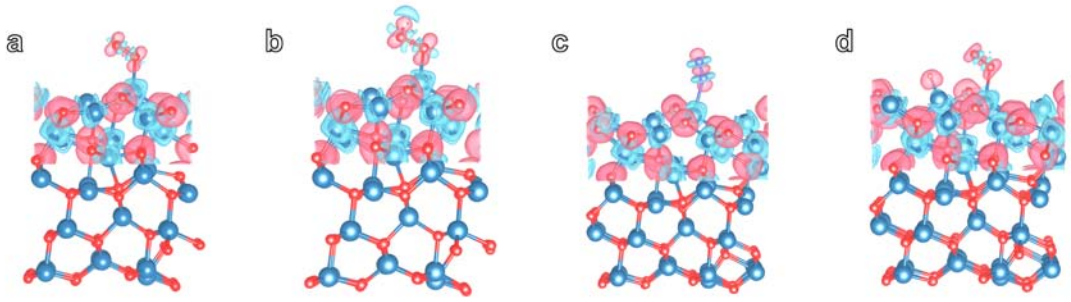

Supplementary Figure 34. Difference charge density for various species adsorbed on ER-ZnO. **a**, $*O_2$. **b,** *OOH. **c,** $*N_2$. **d,** $*O_2$-*H. Red and bule spheres means charge accumulation and depletion, respectively.

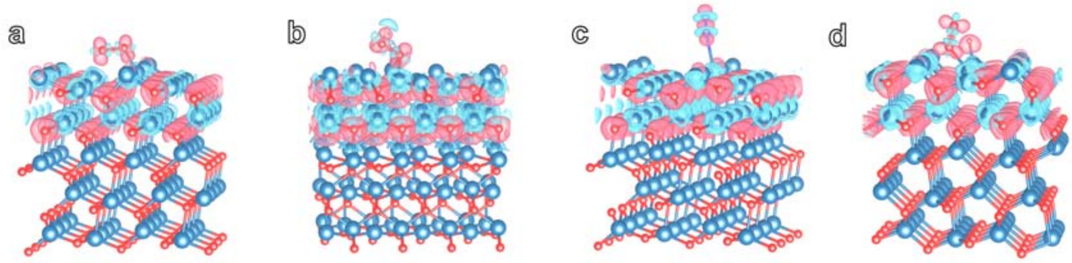

Supplementary Figure 35. Difference charge density for various species adsorbed on ZnO. **a**, $*O_2$. **b,** *OOH. **c,** $*N_2$. **d,** $*O_2$-*H. Red and bule spheres means charge accumulation and depletion, respectively.

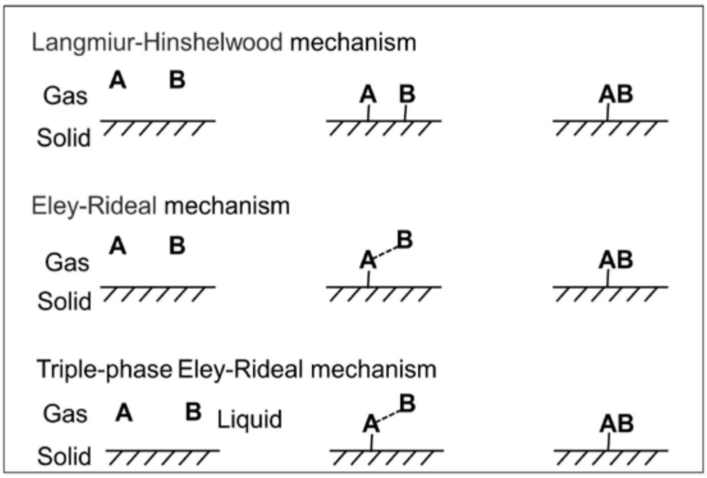

Supplementary Figure 36. The schematic diagrams of L-H mechanism, E-R mechanism and triple-phase E-R mechanism.

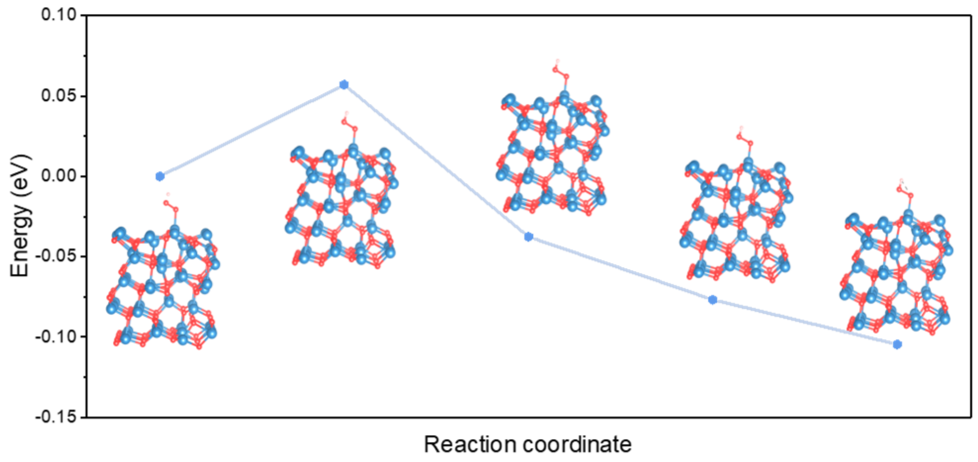

Supplementary Figure 37. Minimum energy path (MEP) of E-R mechanism for ER-ZnO involved in $*O_2$ protonation for two-electron ORR pathway ($*O_2 + H^+ \rightarrow$ *OOH). Inset is the optimized geometry structures of images on the pathway including initial, transition and final states.

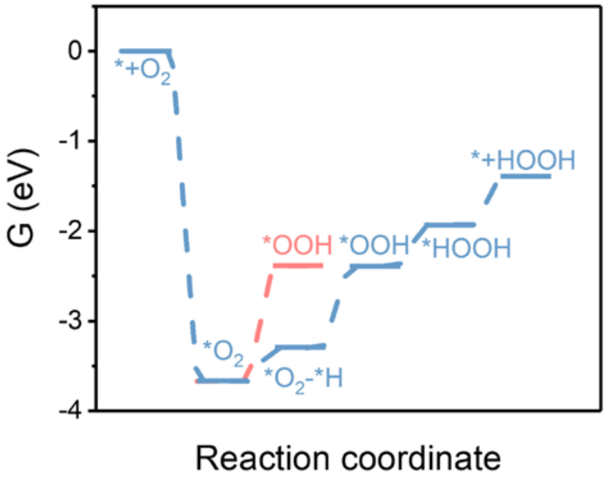

Supplementary Figure 38. The free energy diagram of ORR pathway on the reference ZnO. The two-electron ORR reaction pathway was simulated for L-H (blue line) and E-R mechanism (red line). The *OOH and *O_2-*H coordinate represented the key intermidate step for E-R and L-H mechanism, respectivity. The free energy change demonstrated the reaction difficulty and selectivity.

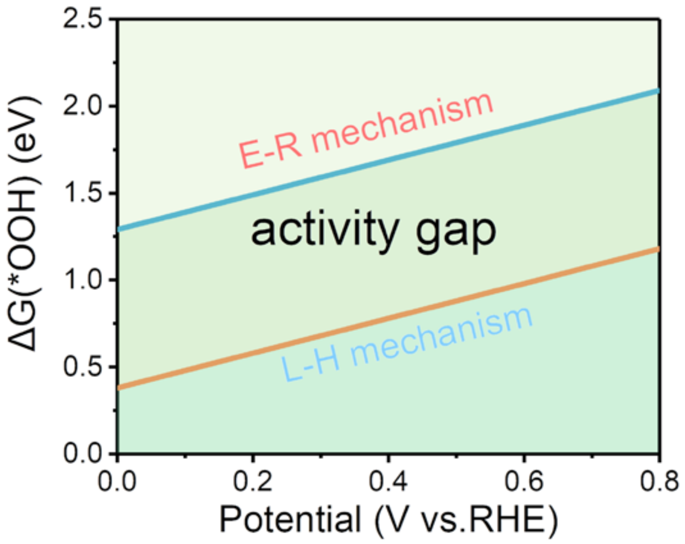

Supplementary Figure 39. The kinetic barrier of E-R mechanism and L-H mechanism on the reference ZnO. The ΔG (*OOH) was calculated according to the free energy change with changing potentials. The activity gap reflected the kinetic barrier between L-H mechanism and E-R mechanism.

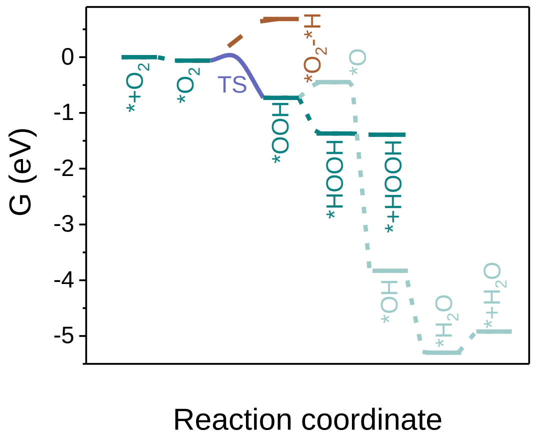

Supplementary Figure 40. The free energy diagrams of two- and four-electron pathways for ER-ZnO. The reaction pathway was simulated for two- (dark green line) and four-electron (light green line) ORR. The $*O_2$-*H coordinate (brown line) represented the L-H mechanism pathway. The free energy change reflected the selectivity for ER-ZnO between L-H and E-R mechanism, as well as two- and four-electron pathways.

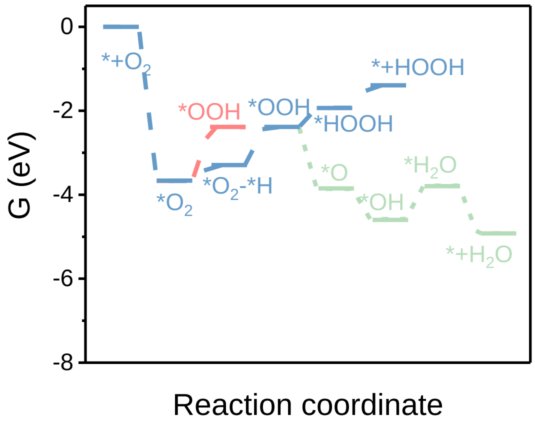

Supplementary Figure 41. The free energy diagrams of two- and four-electron pathways for ZnO. The reaction pathway was simulated for two- (blue line) and four-electron (light green line) ORR. The *OOH coordinate (red line) represented the E-R mechanism pathway. The free energy change reflected the selectivity for reference ZnO between L-H and E-R mechanism, as well as two- and four-electron pathways.

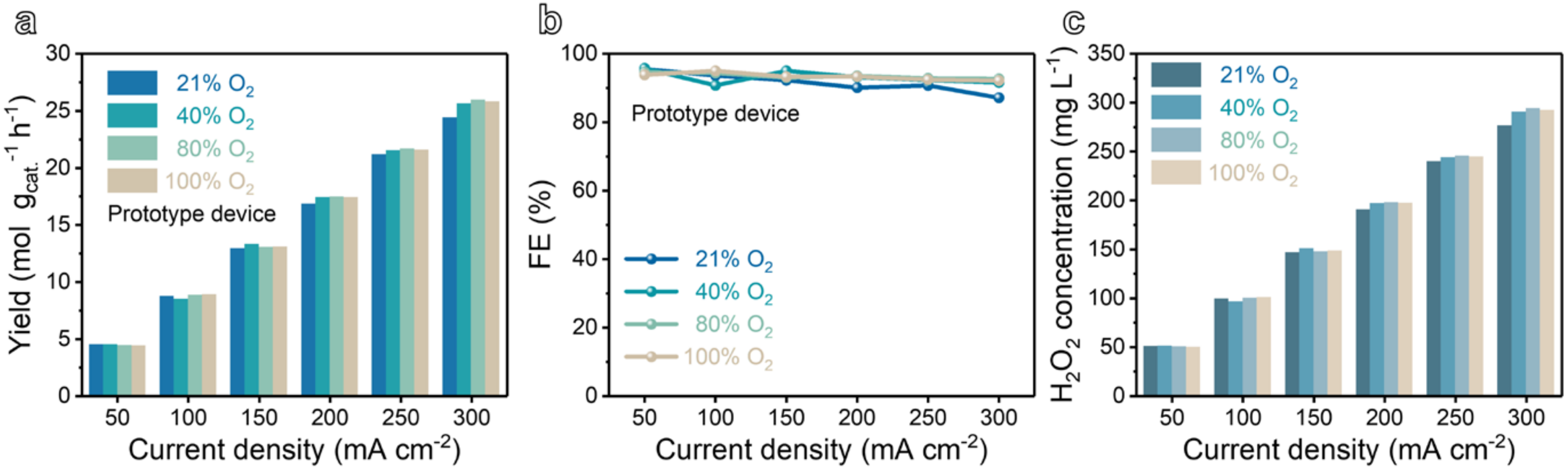

Supplementary Figure 42. **The ORR performances of prototype device in mixed O_2 media**, **a**, yield rates. **b,** Faradaic efficiencies. **c,** H_2O_2 concentrations.

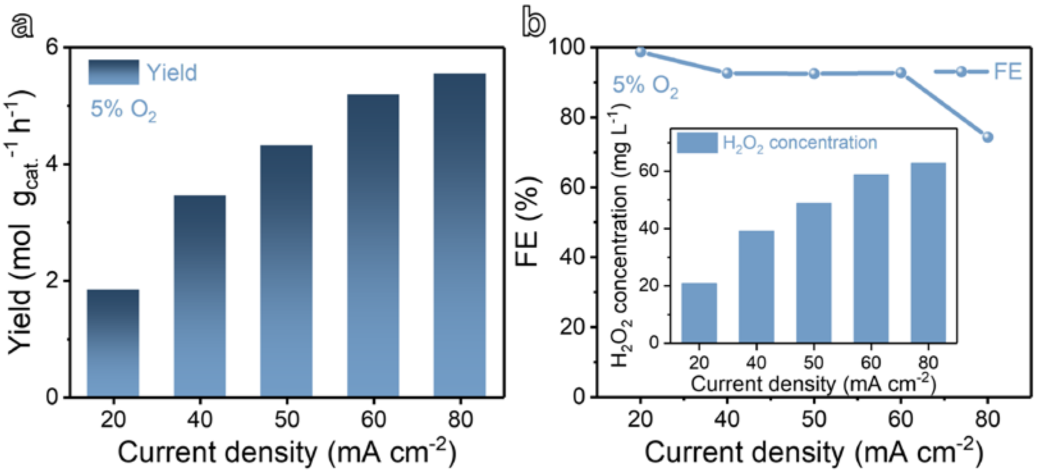

Supplementary Figure 43. **The ORR activity of ER-ZnO catalyst in 5% O_2 a**, H_2O_2 yields tested in flow cells. **b**, H_2O_2 Faradaic efficiencies tested in flow cells, inset with the corresponding H_2O_2 concentration.

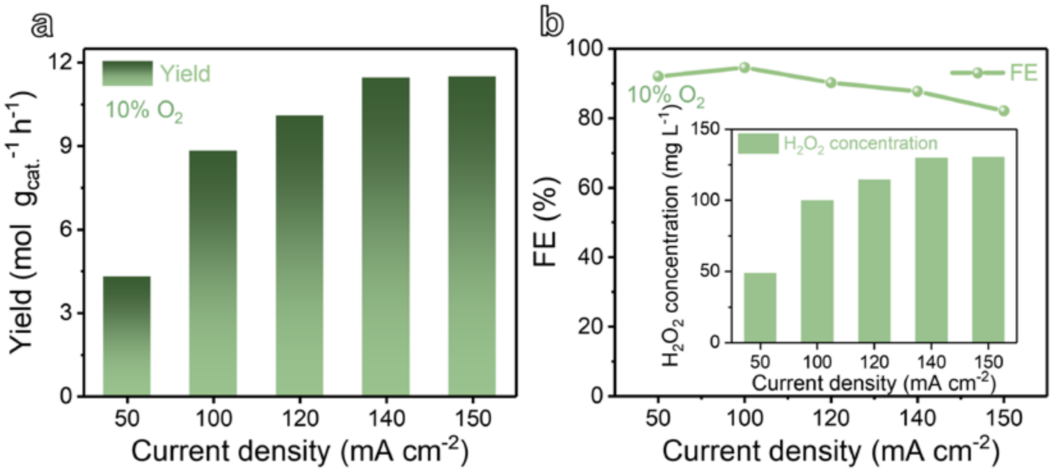

Supplementary Figure 44. **The ORR activity of ER-ZnO catalyst in 10% O_2, a**, H_2O_2 yields tested in flow cells. **b**, H_2O_2 Faradaic efficiencies tested in flow cells, inset with the corresponding H_2O_2 concentrations.

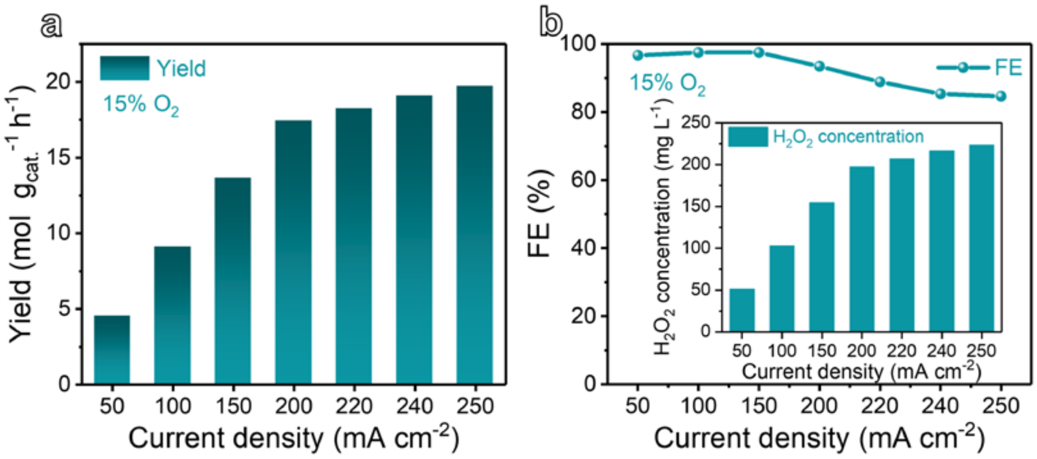

Supplementary Figure 45. **The ORR activity of ER-ZnO catalyst in 15% O_2, a**, H_2O_2 yields tested in flow cells. **b**, H_2O_2 Faradaic efficiencies tested in flow cells, inset with the corresponding H_2O_2 concentrations.

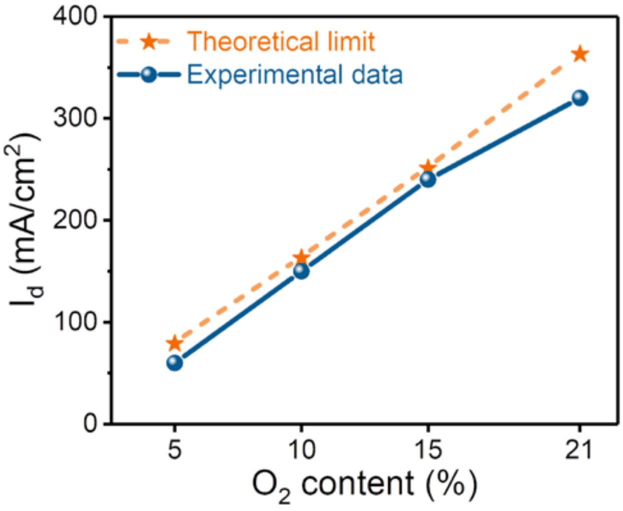

Supplementary Figure 46. The limiting diffusion current densities of ER-ZnO catalyst in low O_2 contents.

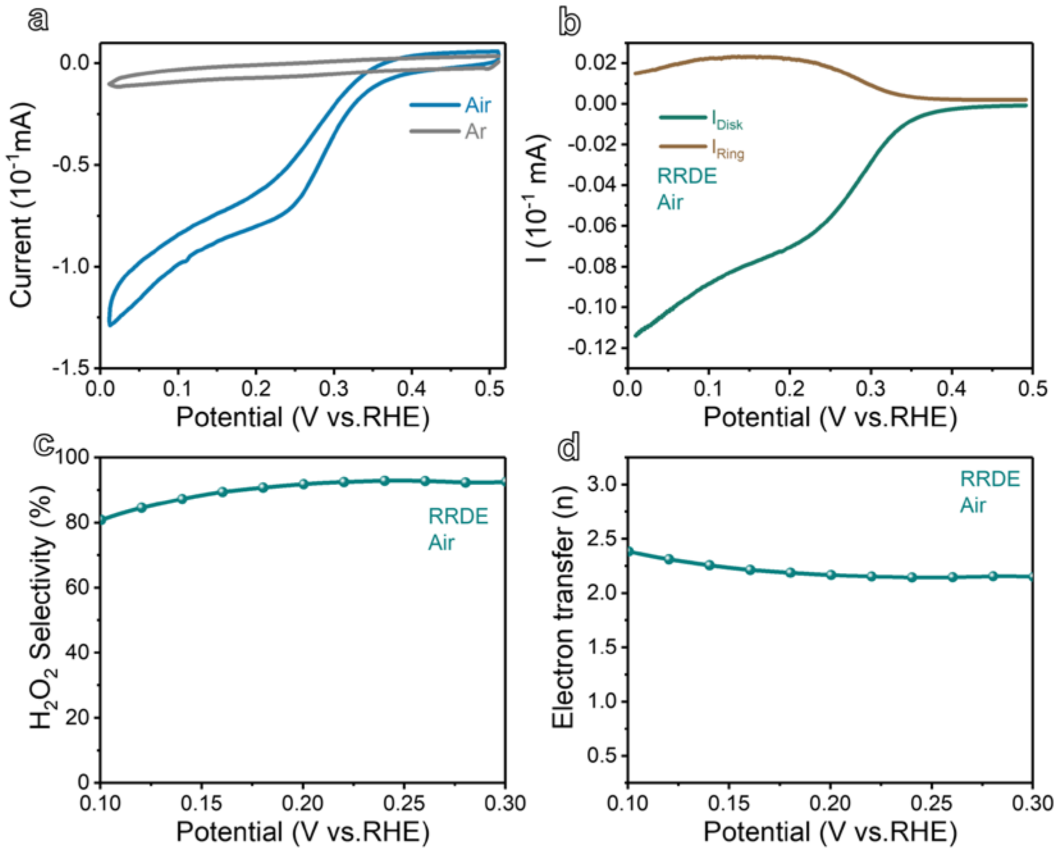

Supplementary Figure 47. **Electrochemical characterizations of ER-ZnO in natural air in RRDE system. a**, The CV curves in Ar and air media. **b,** The LSV polarization curve, and the detected H_2O_2 current with a ring constant potential of 1.2 V. **c**, **d**, the corresponding H_2O_2 molar fraction selectivity and electron transfer number (*n*). The electrochemical characterization in RRDE system was operated in the same condition with mixed dioxygen, except for employing natural air as oxygen source.The H_2O_2 molar fraction selectivity and electron transfer number (*n*) was calculated according the LSV polarization curve.

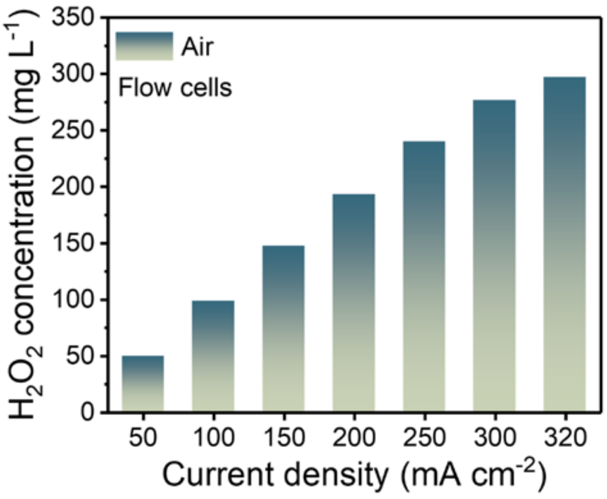

Supplementary Figure 48. The H_2O_2 concentration of ER-ZnO in prototype device in natural air. The prototype device was conducted to produce H_2O_2 in natural air. The electrode was prepared with ER-ZnO catalyst for the same procedure as flow-type cells. The H_2O_2 was accumulated in a glass bottle and H_2O_2 concentration was detected by using UV-vis spectra with $TiOSO_4$ chromogenic reagent.

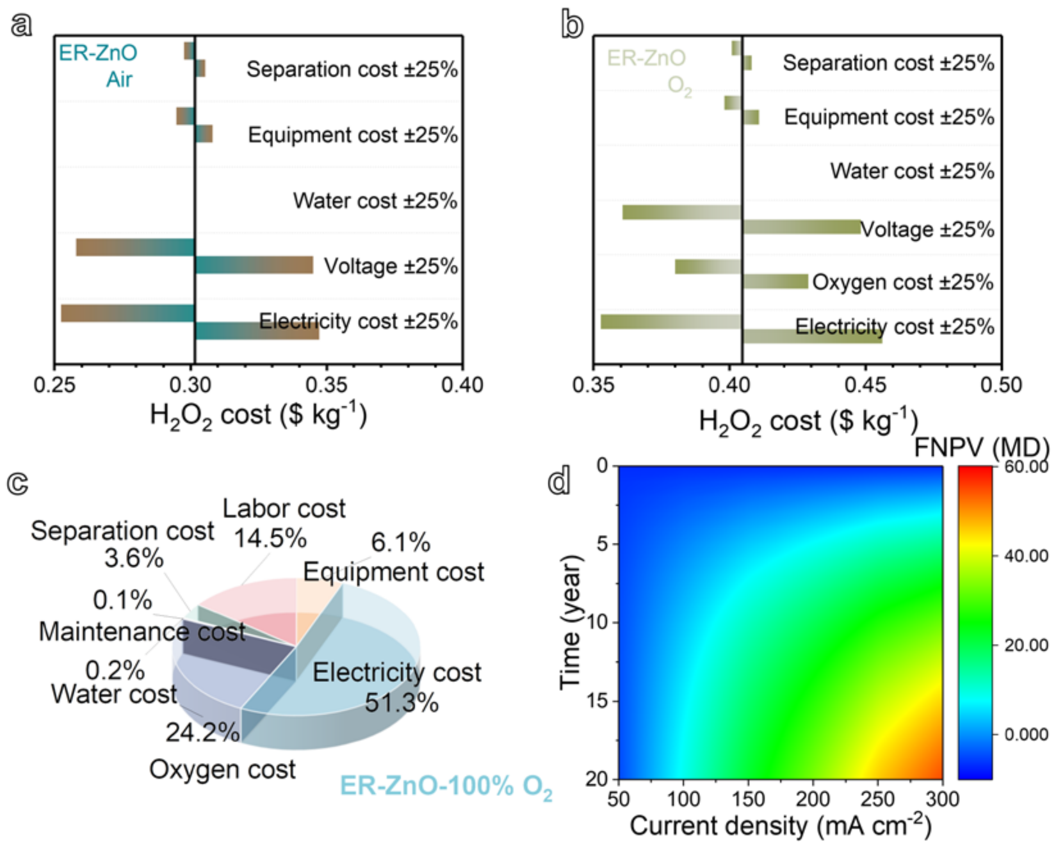

Supplementary Figure 49. The technoeconomic analyses for ER-ZnO in 100% O_2 and air. a, the single variable sensitivity analysis for H_2O_2 production cost by using ER-ZnO catalyst in air. **b**, the single variable sensitivity analysis for H_2O_2 production cost by using ER-ZnO catalyst in 100% O_2 (pure dioxygen). **c**, the proportion of H_2O_2 production cost by using ER-ZnO catalyst in 100% O_2 (pure dioxygen). **d**, Financial net present value analysis of ER-ZnO in 100% O_2 (pure dioxygen).

4. Supplementary Tables

Supplementary Table 1. Zn and O element percentages in ZnO and ER-ZnO calculated from overall XPS surveys.

Material	Zn Atomic %	O Atomic %	Zn vacancy %
ZnO	49.62	50.38	1.51
ER-ZnO	47.72	52.28	8.72

Supplementary Table 2. EXAFS fitting results of Zn-foil, ZnO and ER-ZnO

Material	shell	CN	R(Å)	ΔE_0 (eV)	σ^2 (10^{-3} Å^2)	R factor
Zn-foil	Zn-Zn	6	2.64366	-0.390	0.01325	0.0095565
ZnO	Zn-O	4.006	1.95679	-3.200	0.00614	0.0184869
	Zn-Zn	12.083	3.23427		0.01318	
ER-ZnO	Zn-O	3.906	1.96845	-2.040	0.00281	0.0097350
	Zn-Zn	11.904	3.25917		0.01687	

NOTE: CN: coordination number; R: bond lengths between central atoms and surrounding coordination atoms; σ^2: Debye-Waller factor to account for both thermal and structural disorders; ΔE_0: the difference of the zero kinetic energy value between the sample and theoretical model; R factor is used to measure the goodness of the fitting.

Supplementary Table 3. The onset potentials of ER-ZnO and ZnO in flow cell and RRDE systems, respectively.

Onset potentials (V)	21% O_2	40% O_2	80% O_2	100% O_2	Maximum change
ER-ZnO (Flow cells)	0.416	0.428	0.439	0.441	0.025
ZnO (Flow cells)	0.361	0.380	0.394	0.397	0.036
ER-ZnO (RRDE)	0.344	0.347	0.355	0.358	0.014
ZnO (RRDE)	0.334	0.360	0.364	0.369	0.035

Supplementary Table 4. Zn and O percentages in ER-ZnO after 100 hr-stability test.

Material	Zn Atomic %	O Atomic %	Zn vacancy %
ER-ZnO	47.34	52.66	10.10

Supplementary Table 5. Bader charge transfer of ER-ZnO and reference ZnO with absorbates (*O_2, *N_2, and *O_2/*H in *O_2-*H).

	Charge transfer				
	surface	*O_2	*N_2	*O_2(*O_2-*H)	*H(*O_2-*H)
Zn site (ER-ZnO)	1.17	1.23	1.19	1.12	0.9
Zn site (ZnO)	0.52	1.12	0.7	1.19	0.94

Supplementary Table 6. The ORR activities of ER-ZnO in 21% O_2 (*i.e.*, air) as comparison to reported catalysts.[35-45]

Catalyst	Electrolyte	Loading (mg cm^{-2})	Working area (cm^2)	current density (mA cm^{-2})	Productivity (mol g_{cat}^{-1} h^{-1})	FE (%)	Reference
Ni-N_2O_2/C	0.1 M KOH	0.2	/	70	5.9	91	Angew. Chem. Int. Ed. 2020, 59 (31), 13057-13062.
Co SACs	0.5 M NaCl	0.2	/	50	3.4	58	Energy Environ. Sci. 2021,14, 5444-5456
TRGO-1100	0.1 M KOH	0.28	7	20	2.14	94	Environ. Res. 2022, 204, 112327.
CoS_2/MWCNT	0.05 M Na_2SO_4	2	3	100	0.474	/	J. Electroanal. Chem. 2018, 808, 364-371.
Co-NC/$MXenes_{40}$	0.5 M H_2SO_4	0.2	4	124.6	3.78	90	Appl. Catal. B- Environ. 2022, 317, 121737.
CoPc/CNT	0.1 M H_2SO_4	0.4	12	40	3.71	/	Chinese J. Catal. 2022, 43 (5), 1238-1246.
CFP@MnO_2-Fe_3O_4/C	0.1M Na_2SO_4	0.2	2	5	1.71	/	J. Hazard. Mater. 2021, 412, 125208.
CoSxPy/MWCNTs	0.05 M Na_2SO_4	2	20	40	0.165	60	Appl. Catal. B- Environ. 2019, 247, 191-199.
Co HSACs	0.5 M KOH	0.5	1	300	10	90	Nat Commun 14, 1426 (2023).
graphite	0.1 M Na_2SO_4	0.5625	16	0.75	0.00017	55	Journal of Water Process Engineering 52, 103596 (2023).
NbN_3-(O)C_3N_4/OCNT	0.1 m KOH	1	1	110	2.5	90	ACS Applied Materials & Interfaces 15, 10718-10725 (2023).
ER-ZnO (this work)	0.6 M K_2SO_4	0.2	1	300	24.4	89.3	This work

Supplementary Table 7. The input of streams for techno-economic analyses.

stream	Components	Temperature (℃)	Pressure (atm)	Flow (kg/hr)
Anode	0.6 M K_2SO_4	20	1	8000
Cathode	0.6 M K_2SO_4	20	1	8000
21% O_2 (Air)	-	20	1	15000

Supplementary Table 8 Simulated data of ER-ZnO and ZnO in 21% O_2 (air).

Current densities (A/cm^2)	Potential (V)	material	FE (%)	H_2O_2 production (70wt%, kg/hr)	H_2O consumption of anode (kg/h)	H_2O consumption of cathode (kg/h)	O_2 consumption of cathode (kg/h)	Electrolytic power (kW)	Current (A)	Electrolytic area (m^2)	Anode cycle power (kW)	Cathode cycle power (kW)	O_2 cycle power (kW)	Electrolyte cooling power of Anode (kW)	Electrolyte cooling power of cathode (kW)	Cooling power of H_2O_2(kW)	Cooling power of O_2 (kW)	Membrane separation power (kW)
-0.05	-2.28	ER-ZnO	92.9	421	175	114	0	1140	500000	1000	0.993	1.032	21.594	175.675	181.648	7.52	21.594	3.518
-0.1	-2.5	ER-ZnO	93	841	367	226	0	2500	1000000	1000	1.007	1.088	21.594	303.424	329.94	27.257	21.594	6.877
-0.15	-2.73	ER-ZnO	92.6	1258	573	340	0	4095	1500000	1000	1.012	1.141	21.594	365.604	426.475	52.704	21.594	9.927
-0.2	-2.87	ER-ZnO	90	1631	781	422	0	5740	2000000	1000	1.015	1.183	21.594	390.409	486.958	78.173	21.594	12.495
-0.25	-3.1	ER-ZnO	90.4	2046	992	531	0	7750	2500000	1000	1.016	1.23	21.594	405.583	540.159	108.658	21.594	15.231
-0.3	-3.22	ER-ZnO	87.2	2368	1204	579	0	9660	3000000	1000	1.018	1.267	21.594	416.004	593.706	138.548	21.594	17.217
-0.05	-2.95	ZnO	84.8	385	177	90	0	1475	500000	1000	0.998	1.036	21.594	230.656	240.298	9.075	21.594	3.233
-0.1	-3.13	ZnO	85.1	774	378	183	0	3130	1000000	1000	1.01	1.088	21.594	348.647	386.215	29.422	21.594	6.299
-0.15	-3.38	ZnO	74.3	1010	591	174	0	5070	1500000	1000	1.016	1.123	21.594	398.74	482.355	47.921	21.594	8.152
-0.2	-3.53	ZnO	66.9	1213	801	142	0	7060	2000000	1000	1.017	1.151	21.594	414.554	544.996	65.014	21.594	9.641
-0.25	-3.71	ZnO	62.8	1424	1014	114	0	9275	2500000	1000	1.018	1.178	21.594	425.45	606.254	84.962	21.594	11.143
-0.3	-3.88	ZnO	51.5	1397	1367	164	0	11640	3000000	1000	1.027	1.19	21.594	498.9	720.865	99.094	21.594	11.034

Supplementary Table 9. Simulated data of ER-ZnO in 100% O_2 (pure dioxygen).

Current densities (A/cm^2)	Potential (V)	material	FE (%)	H_2O_2 production (70wt%, kg/hr)	H_2O consumption of anode (kg/h)	H_2O consumption of cathode (kg/h)	O_2 consumption of cathode (kg/h)	Electrolytic power (kW)	Current (A)	Electrolytic area (m^2)	Anode cycle power (kW)	Cathode cycle power (kW)	O_2 cycle power (kW)	Electrolyte cooling power of Anode (kW)	Electrolyte cooling power of cathode (kW)	Cooling power of H_2O_2(kW)	Cooling power of O_2 (kW)	Membrane separation power (kW)
-0.05	-2.75	ER-ZnO	94.7	429	177	120	291	1375	500000	1000	0.999	1.039	224.643	224.397	232.134	9.777	251.341	3.602
-0.1	-2.9	ER-ZnO	95.3	865	375	244	583	2900	1000000	1000	1.009	1.096	231.54	336.69	369.408	31.398	319.109	7.009
-0.15	-3.06	ER-ZnO	95.1	1293	583	363	874	4590	1500000	1000	1.014	1.147	233.834	385.054	451.994	57.391	357.398	10.178
-0.2	-3.21	ER-ZnO	93.8	1702	793	469	1157	6420	2000000	1000	1.017	1.194	233.969	405.166	508.443	85.052	381.307	12.947
-0.25	-3.31	ER-ZnO	91.2	2070	1001	549	1427	8275	2500000	1000	1.017	1.234	233.34	414.305	555.159	113.118	399.236	15.279
-0.3	-3.43	ER-ZnO	92.5	2513	1221	677	1724	10290	3000000	1000	1.019	1.286	233.247	428.109	614.739	151.94	422.885	18.062

Supplementary Table 10. FNPV ($) analysis of ER-ZnO at different current densities in 21% O_2 (air).

Time (year)	-0.30 A cm^{-2}	-0.25 A cm^{-2}	-0.2 A cm^{-2}	-0.15 A cm^{-2}	-0.10 A cm^{-2}	-0.05 A cm^{-2}
0	-6948946.791	-6948946.791	-6948946.791	-6948946.791	-6948946.791	-6948946.791
1	-1810904.133	-2488511.007	-3443052.513	-4324908.445	-5342263.272	-6390284.557
2	2829104.289	1506157.536	-357471.1185	-2079189.844	-4065454.03	-6111590.825
3	7231153.127	5293596.965	2564171.026	42582.97315	-2866452.03	-5863175.025
4	11406573.79	8883675.332	5329680.551	2046312.187	-1741552.167	-5643595.352
5	15366158.09	12285790.78	7946492.343	3937618.921	-687225.7659	-5451478.656
6	19120183.96	15508893.94	10421687.25	5721856.627	299887.8129	-5285517.178
7	22678439.89	18561509.18	12762008.93	7404123.831	1222989.181	-5144465.429
8	26050248.26	21451754.99	14973879.91	8989276.269	2085126.537	-5027137.233
9	29244487.52	24187363.25	17063416.9	10481938.46	2889202.932	-4932402.897
10	32269613.34	26775697.65	19036445.33	11886514.69	3637983.171	-4859186.522
11	35133678.76	29223771.22	20898513.22	13207199.53	4334100.406	-4806463.445
12	37844353.3	31538263.06	22654904.41	14447987.82	4980062.399	-4773257.792
13	40408941.3	33725534.2	24310651.12	15612684.15	5578257.493	-4758640.164
14	42834399.26	35791642.76	25870545.95	16704911.95	6130960.305	-4761725.416
15	45127352.42	37742358.4	27339153.27	17728122.1	6640337.132	-4781670.554
16	47294110.53	39583176.02	28720820.1	18685601.15	7108451.117	-4817672.727
17	49340682.88	41319328.86	30019686.47	19580479.16	7537267.157	-4868967.313
18	51272792.6	42955800.94	31239695.26	20415737.12	7928656.583	-4934826.102
19	53095890.29	44497338.99	32384601.59	21194214.1	8284401.615	-5014555.561
20	54815167.01	45948463.65	33457981.77	21918613.94	8606199.604	-5107495.182

Supplementary Table 11. FNPV ($) analysis of ER-ZnO at 0.3 A cm^{-2} in 100% O_2 (pure dioxygen) and 21% O_2 (air).

Time (year)	21% O_2 (air)	100% O_2
0	-6948946.791	-6948946.791
1	-1810904.133	-2557262.096
2	2829104.289	1371929.22
3	7231153.127	5097009.384
4	11406573.79	8627697.976
5	15366158.09	11973251.74
6	19120183.96	15142486.61
7	22678439.89	18143798.74
8	26050248.26	20985184.43
9	29244487.52	23674259.25
10	32269613.34	26218276.08
11	35133678.76	28624142.45
12	37844353.3	30898436.95
13	40408941.3	33047424.91
14	42834399.26	35077073.3
15	45127352.42	36993064.97
16	47294110.53	38800812.14
17	49340682.88	40505469.31
18	51272792.6	42111945.52
19	53095890.29	43624916.07
20	54815167.01	45048833.59

5. Supplementary Reference

1. Perdew, Burke & Ernzerhof. Generalized gradient approximation made simple. *Phys. Rev. Lett.* **77**, 3865-3868 (1996).
2. Perdew, Burke & Wang. Generalized gradient approximation for the exchange-correlation hole of a many-electron system. *Phys. Rev., B Condens. Matter.* **54**, 16533-16539 (1996).
3. Blochl, P. E. Projector augmented-wave method. *Phys. Rev., B Condens. Matter.* **50**, 17953-17979 (1994).
4. Kresse, G. & Joubert, D. From ultrasoft pseudopotentials to the projector augmented-wave method. *Phys. Rev., B Condens. Matter.* **59**, 1758-1775 (1999).
5. Chang, H., Liau, J.-S., Ho, C.-D. & Wang, W.-H. Simulation of membrane distillation modules for desalination by developing user's model on Aspen Plus platform. *Desalination* **249**, 380-387 (2009).
6. Chu, S., Cui, Y. & Liu, N. The path towards sustainable energy. *Nat. Mater.* **16**, 16-22 (2017).
7. Zuo, G. Z., Wang, R., Field, R. & Fane, A. G. Energy efficiency evaluation and economic analyses of direct contact membrane distillation system using Aspen Plus. *Desalination* **283**, 237-244 (2011).
8. Bahmanyar, A., Asghari, M. & Khoobi, N. Numerical simulation and theoretical study on simultaneously effects of operating parameters in direct contact membrane distillation. *Chem. Eng. Process.* **61**, 42-50 (2012).
9. Hitsov, I., et al. Modelling approaches in membrane distillation: a critical review. *Sep. Purif. Technol.* **142**, 48-64 (2015).
10. Von Kurnatowski, M. & Bortz, M. Modeling and multi-criteria optimization of a process for H_2O_2 electrosynthesis. *Processes* **9**, (2021).
11. Xia, C., et al. Direct electrosynthesis of pure aqueous H_2O_2 solutions up to 20% by weight using a solid electrolyte. *Science* **366**, 226–231 (2019).

12. Xia, Y., et al. Highly active and selective oxygen reduction to H_2O_2 on boron-doped carbon for high production rates. *Nat. Commun.* **12**, 4225 (2021).
13. Zhang, X., Xia, Y., Xia, C. & Wang, H. Insights into practical-scale electrochemical H_2O_2 synthesis. *Trends in Chemistry* **2**, 942-953 (2020).
14. Huang, Z., Grim, R. G., Schaidle, J. A. & Tao, L. The economic outlook for converting CO_2 and electrons to molecules. *Energy Environ. Sci.* **14**, 3664-3678 (2021).
15. Xia, C., et al. Continuous production of pure liquid fuel solutions via electrocatalytic CO_2 reduction using solid-electrolyte devices. *Nat. Energy* **4**, 776-785 (2019).
16. Chen, Y. J., et al. Single-atom catalysts: Synthetic strategies and electrochemical applications. *Joule* **2**, 1242-1264 (2018).
17. Lang, R., et al. Non defect-stabilized thermally stable single-atom catalyst. *Nat. Commun.* **10**, 234 (2019).
18. Wang, A. Q., Li, J. & Zhang, T. Heterogeneous single-atom catalysis. *Nat. Rev. Chem.* **2**, 65-81 (2018).
19. Cheng, N., Zhang, L., Doyle-Davis, K. & Sun, X. L. Single-atom catalysts: From design to application. *Electrochem. Energy Rev.* **2**, 539-573 (2019).
20. Kaiser, S. K., et al. Single-atom catalysts across the periodic table. *Chem. Rev.* **120**, 11703-11809 (2020).
21. Favaro, M., Kong, H. & Gottesman, R. In situ and operando Raman spectroscopy of semiconducting photoelectrodes and devices for photoelectrochemistry. *J. Phys. D: Appl. Phys.* **57**, 103002 (2023).
22. Li, H. Y., et al. Operando electrochemical X-ray diffraction and raman spectroscopic studies revealing the alkali-metal ion intercalation mechanism in prussian blue analogues. *J. Phys. Chem. Lett.* **13**, 479-485 (2022).
23. Dong, J.-C., et al. Direct In Situ Raman Spectroscopic Evidence of Oxygen Reduction Reaction Intermediates at High-Index Pt(hkl) Surfaces. *J. Am. Chem. Soc.* **142**, 715-719 (2020).

24. Shan, W. Y., et al. In Situ Surface-Enhanced Raman Spectroscopic Evidence on the Origin of Selectivity in CO2 Electrocatalytic Reduction. *ACS Nano* **14**, 11363-11372 (2020).
25. Zhao, Y. L., et al. Identification of M-NH2-NH2 Intermediate and Rate Determining Step for Nitrogen Reduction with Bioinspired Sulfur-Bonded FeW Catalyst. *Angew. Chem. Int. Edit.* **60**, 20331-20341 (2021).
26. Cui, X. Y., Tang, C. & Zhang, Q. A review of electrocatalytic reduction of dinitrogen to ammonia under ambient conditions. *Adv. Energy Mater.* **8**, 1800369 (2018).
27. Tang, C., et al. Coordination tunes selectivity: Two-electron oxygen reduction on high-loading molybdenum single-atom catalysts. *Angew. Chem. Int. Edit.* **59**, 9171-9176 (2020).
28. Ye, T.-N., et al. Vacancy-enabled N_2 activation for ammonia synthesis on an Ni-loaded catalyst. *Nature* **583**, 391-395 (2020).
29. Quan, J., et al. Vibration-driven reaction of CO_2 on Cu surfaces via Eley–Rideal-type mechanism. *Nat. Chem.* **11**, 722-729 (2019).
30. Weinberg, W. H. Eley-Rideal surface chemistry: direct reactivity of gas phase atomic hydrogen with adsorbed species. *Acc. Chem. Res.* **29**, 479-487 (1996).
31. Kulkarni, A., Siahrostami, S., Patel, A. & Nørskov, J. K. Understanding catalytic activity trends in the oxygen reduction reaction. *Chem. Rev.* **118**, 2302-2312 (2018).
32. Wang, J., et al. Quantitative kinetic analysis on oxygen reduction reaction: A perspective. *Nano Mater. Sci.* **3**, 313-318 (2021).
33. Zheng, M., et al. Electrocatalytic CO_2-to-C_2^+ with ampere-level current on heteroatom-engineered copper via tuning *CO intermediate coverage. *J. Am. Chem. Soc.* **144**, 14936-14944 (2022).
34. Zha, Q. X. *Introduction to electrode process kinetics*, Ch. 9 (Science Press, 2002).

35. Dong, P., et al. Heterogeneous electro-Fenton catalysis with self-supporting CFP@MnO_2-Fe_3O_4/C cathode for shale gas fracturing flowback wastewater. *J. Hazard. Mater.* **412**, 125208 (2021).

36. Fan, W., et al. Rational design of heterogenized molecular phthalocyanine hybrid single-atom electrocatalyst towards two-electron oxygen reduction. *Nat. Commun.* **14**, 1426 (2023).

37. Huang, X., et al. Nb_2CT_x MXenes functionalized Co-NC enhancing electrochemical H_2O_2 production for organics degradation. *Appl. Catal. B: Environ.* **317**, 121737 (2022).

38. Li, D. D., et al. External-shell oxygen enabling the local environment modulation of unsaturated NbN_3 for efficient electrosynthesis of hydrogen peroxide. *ACS Appl. Mater. Interfaces* **15**, 10718-10725 (2023).

39. Li, W., et al. Thermal reduced graphene oxide enhanced in-situ H_2O_2 generation and electrochemical advanced oxidation performance of air-breathing cathode. *Environ. Res.* **204**, 112327 (2022).

40. Liu, C., Chu, Y., Wang, R. & Fan, J. Preparation of lotus-leaf-like carbon cathode for the electro-Fenton oxidation process: hydrogen peroxide production, various organics degradation and printing wastewater treatment. *J. Water Process. Eng.* **52**, 103596 (2023).

41. Ridruejo, C., et al. On-site H_2O_2 electrogeneration at a CoS_2-based air-diffusion cathode for the electrochemical degradation of organic pollutants. *J. Electroanal. Chem.* **808**, 364-371 (2018).

42. Wang, Y. L., et al. High-efficiency oxygen reduction to hydrogen peroxide catalyzed by nickel single-atom catalysts with tetradentate N_2O_2 coordination in a three-phase flow cell. *Angew. Chem. Int. Edit.* **59**, 13057-13062 (2020).

43. Ye, Z. H., et al. Enhanced electrocatalytic production of H_2O_2 at Co-based air-diffusion cathodes for the photoelectro-Fenton treatment of bronopol. *Appl. Catal. B: Environ.* **247**, 191-199 (2019).

44. Yu, Z. X., et al. Interfacial engineering of heterogeneous molecular electrocatalysts using ionic liquids towards efficient hydrogen peroxide production. *Chinese J. Catal.* **43**, 1238-1246 (2022).

45. Zhao, Q. L., et al. Approaching a high-rate and sustainable production of hydrogen peroxide: oxygen reduction on Co-N-C single-atom electrocatalysts in simulated seawater. *Energy Environ. Sci.* **14**, 5444-5456 (2021).
