# Supplementary material for: Single-zinc vacancy unlocks high-rate H2O2 electrosynthesis from mixed dioxygen beyond Le Chatelier principle
Source: Nat Commun. 2024 May 16;15:4157. doi: 10.1038/s41467-024-48256-7 (PMC11098813; doi:10.1038/s41467-024-48256-7)
Supplement: Supplementary file 3 — Description of Additional Supplementary Information [file 41467_2024_48256_MOESM3_ESM.pdf]

Supplementary Movie 1. The prototype device for pollutant degradation of rhodamine B. The solution flows through the prototype device operating in atmospheric air at 300 mA cm^{-2}, where the color of rhodamine B solution fades rapidly.
